# Rapid and dynamic processing of face pareidolia in the human brain

**DOI:** 10.1038/s41467-020-18325-8

**Published:** 2020-09-09

**Authors:** Susan G. Wardle, Jessica Taubert, Lina Teichmann, Chris I. Baker

**Affiliations:** 1 Section on Learning and Plasticity, Laboratory of Brain and Cognition, National Institute of Mental Health, Bethesda, MD USA; 2 Section on Neurocircuitry, Laboratory of Brain and Cognition, National Institute of Mental Health, Bethesda, MD USA; 3 Department of Cognitive Science, Macquarie University, Sydney, NSW Australia

**Keywords:** Perception, Extrastriate cortex, Object vision

## Abstract

The human brain is specialized for face processing, yet we sometimes perceive illusory faces in objects. It is unknown whether these natural errors of face detection originate from a rapid process based on visual features or from a slower, cognitive re-interpretation. Here we use a multifaceted approach to understand both the spatial distribution and temporal dynamics of illusory face representation in the brain by combining functional magnetic resonance imaging and magnetoencephalography neuroimaging data with model-based analysis. We find that the representation of illusory faces is confined to occipital-temporal face-selective visual cortex. The temporal dynamics reveal a striking evolution in how illusory faces are represented relative to human faces and matched objects. Illusory faces are initially represented more similarly to real faces than matched objects are, but within ~250 ms, the representation transforms, and they become equivalent to ordinary objects. This is consistent with the initial recruitment of a broadly-tuned face detection mechanism which privileges sensitivity over selectivity.

## Abstract (web summary)

The human brain is specialised for face processing, yet sometimes objects are perceived as illusory faces. Here, the authors show that illusory faces are initially represented similarly to real faces, but the representation quickly transforms into one equivalent to ordinary objects.

## Introduction

Humans are incredibly skilled at both detecting and recognizing faces, with a significant region of cortex dedicated to face processing^1^. Despite this expertise, sometimes we spontaneously perceive faces where there are none—for example in inanimate objects, such as in a tree or a piece of fruit. This phenomenon, known as face pareidolia, can be conceptualized as a natural error of the face-detection system and has recently been demonstrated behaviorally in macaque monkeys^2,3^, suggesting that the perception of illusory faces arises from a fundamental feature of the primate face-detection system, rather than being a uniquely human trait. Despite substantial progress in uncovering the primate face processing system^1,4–7^ it is still not understood what constitutes a face for visual cortex, and what neural mechanism elicits errors of face detection in ordinary objects.

**Fig. 1:**
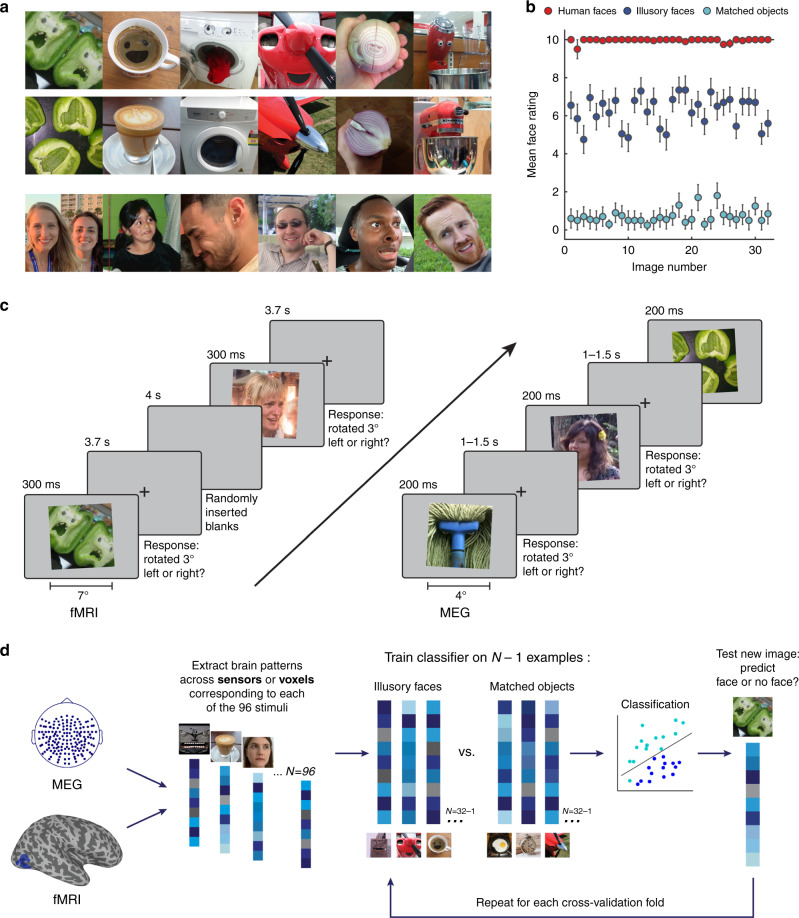
Experimental design and analysis. **a** Example visual stimuli from the set of 96 photographs used in all experiments. The set included 32 illusory faces, 32 matched objects without an illusory face, and 32 human faces. Note that the human face images used in the experiments are not shown in the figure because we do not have the rights to publish them. The original face stimuli used in the experiments are available at the Open Science Framework website for this project: https://osf.io/9g4rz. The human faces shown in this figure are similar photographs taken of lab members who gave permission to publish their identifiable images. See Supplementary Fig. 1 for all 96 visual stimuli. Full resolution versions of the stimuli used in the experiment are available at the Open Science Framework website for this project: https://osf.io/9g4rz. **b** Behavioral ratings for the 96 stimuli were collected by asking *N* = 20 observers on Amazon Mechanical Turk to “Rate how easily can you can see a face in this image” on a scale of 0–10. Illusory faces are rated as more face-like than matched nonface objects. Error bars are ±1 SEM. Source data are provided as a Source data file. **c** Event-related paradigm used for the fMRI (*n* = 16) and MEG (*n* = 22) neuroimaging experiments. In both experiments the 96 stimuli were presented in random order while brain activity was recorded. Due to the long temporal lag of the fMRI BOLD signal, the fMRI version of the experiment used a longer presentation time and longer interstimulus-intervals than the MEG version. To maintain alertness the participants’ task was to judge whether each image was tilted slightly to the left or right (3°) using a keypress (fMRI, mean = 92.5%, SD = 8.6%; MEG, mean = 93.2%, SD = 4.8%). **d** Method for leave-one-exemplar-out cross-decoding. A classifier was trained to discriminate between a given category pair (e.g., illusory faces and matched objects) by training on the brain activation patterns associated with all of the exemplars of each category except one, which was left out as the test data from a separate run for the classifier to predict the category label. This process was repeated across each cross-validation fold such that each exemplar had a turn as the left-out data. Accuracy was averaged across all cross-validation folds.

Here, we combine noninvasive neuroimaging tools with high temporal (MEG) and spatial (fMRI) resolution as well as behavioral ratings and model-based analyses in order to understand how illusory faces are processed in the human brain. Critical to our approach here is the use of a yoked stimulus design. For each illusory face we found a matched object image, which was semantically and visually similar, but which did not contain an illusory face (Fig. 1). The matched set of objects facilitates examination of how the presence of an illusory face modulates the brain’s representation of an object. In terms of the spatial distribution of responses, previous findings suggest a considerable degree of abstraction in the visual selectivity of face-responsive brain regions^5,6,8–11^. The sensitivity of face-selective regions to abstract faces^5,8,9^ suggests these regions are likely sensitive to illusory faces in inanimate objects, but it is an open question whether this sensitivity is specific to face-selective regions, or whether it is widespread throughout category-selective cortex, including regions selective to objects^12,13^. This makes illusory faces in objects particularly interesting in terms of their category membership as they are perceived as both an object and as a face. Importantly, natural examples of illusory faces are visually diverse and do not require any assumptions to be made about the key features that drive the brain’s response to face stimuli. This means that illusory faces are potentially revealing about the behaviorally relevant tuning of the face-detection system.

While understanding the spatial organization of responses to illusory faces will clarify the role of higher-level visual cortex in face perception, identifying the temporal dynamics of how illusory faces are processed is critical to understanding the origin of these face-detection errors. Human faces are rapidly detected by the human brain^12,13^, but it is not known to what extent illusory face perception relies upon the same neural mechanisms. One possibility is that certain arrangements of visual features (such as a round shape) rapidly activate a basic face-detection mechanism, leading to the erroneous perception of a face. Alternatively, illusory face perception may arise from a slower cognitive reinterpretation of visual attributes as facial features, for example as eyes or a mouth. If illusory faces are rapidly processed, it would suggest the face-detection mechanism is broadly tuned and weighted toward high sensitivity at the cost of increased false alarms. Here we exploit the high temporal resolution of MEG in order to distinguish between these alternative accounts in the human brain.

We find that face-selective regions are sensitive to the presence of an illusory face in an inanimate object, but other occipital–temporal category-selective visual regions are not. In addition to this spatially restricted response, we discover a transient and rapidly evolving response to illusory faces. In the first couple of 100 ms, illusory faces are represented more similarly to human faces than their yoked nonface object counterparts are. However, within only 250 ms after stimulus onset, this representation shifts such that illusory faces are indistinguishable from ordinary objects. In order to enhance our understanding of what is driving this early face-like response to illusory faces, we implement a model-based analysis to compare the brain’s response to behavioral ratings of “faceness” and the output of computational models of visual features. We find that the brain’s representation correlates earlier with the visual feature models than the behavioral model, although the behavioral model explained more variance in the brain’s response overall than the computational models. Together, our results demonstrate that that an initial erroneous face-like response to illusory faces is rapidly resolved, with the representational structure quickly stabilizing into one organized by object content rather than by face perception.

## Results

### Recording responses to illusory faces in the human brain

We recorded neuroimaging data (fMRI and MEG) in two separate experiments while human participants viewed 96 photographs including 32 examples of illusory faces in inanimate objects, 32 matched nonface objects, and 32 human faces (Fig. 1a; Supplementary Fig. 1). The nonface objects were yoked to the illusory face examples, so that each illusory face was paired with a matched nonface object that was of the same object identity and as visually similar as possible (see Online methods). The stimulus set was validated by asking workers on Amazon Mechanical Turk (*n* = 20) to “Rate how easily can you can see a face in this image” on a scale of 0–10. Human faces (*M* = 9.96, SD = 0.10), were rated as more face-like than both illusory faces (*t*(31) = 26.15, *p* < 0.001, two-tailed, Bonferroni correction for *k* = 3 comparisons) and matched objects (*t*(31) = 143.57, *p* < 0.001). Importantly, illusory faces (*M* = 6.27, SD = 0.79) were rated as significantly more face-like than matched nonface objects (*M* = 0.70, SD = 0.36), demonstrating that faces are spontaneously perceived in the images we selected (*t*(31) = 39.68, *p* < 0.001; Fig. 1b).

We collected fMRI and MEG data for the same stimulus set to benefit maximally from the higher spatial resolution of fMRI and the finer temporal resolution of MEG (Fig. 1c). Due to the relative temporal sluggishness of the fMRI BOLD response, in the fMRI experiment (*N* = 21), images were shown for 300 ms with a 3.7 s interstimulus interval and additional randomly inserted blank trials of 4 s duration. In the MEG experiment (*N* = 22), images were shown for 200 ms with a 1–1.5 s variable interstimulus interval. In order to maintain alertness, in both experiments participants judged whether the image on each trial was rotated slightly to the left or right by 3°.

We used a multivariate pattern analysis approach^14–17^ to analyze the neuroimaging data and extracted the brain activation patterns associated with viewing each of the 96 visual images (Fig. 1d). For the fMRI data, we extracted the spatial patterns across voxels in response to each of the visual stimuli, providing finer spatial resolution to complement the high temporal resolution of the MEG data. For the MEG experiment, we isolated the neuromagnetic signal from −100 to 1000 ms relative to stimulus onset on each trial and extracted the spatiotemporal patterns across the whole brain across all 160 sensors as a function of time. These patterns across voxels (fMRI) or sensors (MEG) were then used for the specific analyses detailed below.

### Illusory faces modulate responses in face-selective cortex

**Fig. 2:**
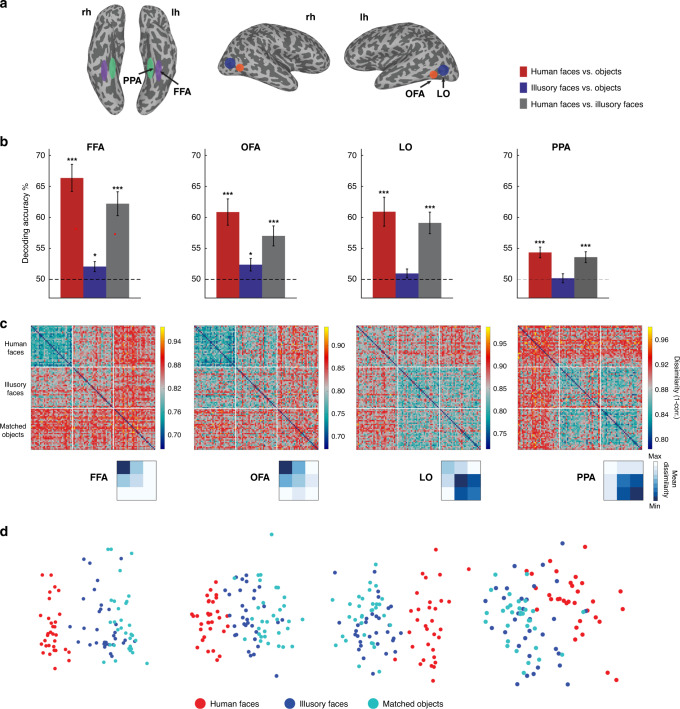
fMRI results showing sensitivity to illusory faces in face-selective cortex. **a** Schematic visualization of the four functional regions of interest; each region was defined individually in each hemisphere of each subject from their functional localizer. **b** Results of cross-decoding (train and test the classifier on brain activity associated with different exemplars so generalization across stimuli is required) the three stimulus categories from four regions of interest. The mean decoding accuracy is shown, averaged over *N* = 16 participants. Asterisks indicate conditions with statistically significant decoding, evaluated using one-sample *t*-tests (one-tailed) and FDR adjusted *p values < 0.05, and ***p values < 0.001 to correct for multiple comparisons. The distinction between human faces and objects with (FFA: *p* = 0.00004, OFA: *p* = 0.0005, LO: *p* = 0.0002, PPA: *p* = 0.0008) or without an illusory face (FFA: *p* = 0.00001, OFA: *p* = 0.0002, LO: *p* = 0.0003, PPA: *p* = 0.0002) can be decoded from activation patterns in all regions. Illusory faces can be discriminated from similar matched objects from activity in FFA (*p* = 0.015) and OFA (*p* = 0.020) only, but not in LO (*p* = 0.11) and PPA (*p* = 0.40). Error bars are SEM. Source data are provided as a Source data file**. c** Representational dissimilarly matrices (96 × 96) for all stimuli for the four regions of interest. The dissimilarity is calculated by taking 1-correlation (Spearman) between the BOLD activation patterns for each pair of stimuli. The colorbar range is scaled to the max and min of the dissimilarity values for each ROI for visualization. White lines indicate stimulus category boundaries. Insets show 3 × 3 matrices for each ROI averaged by category, excluding the diagonal. Source data are provided as a Source data file. **d** Visualization of the dissimilarity matrices in (**c**) using multidimensional scaling. The first two dimensions following MDS are plotted, each of the points representing the 96 stimuli is colored according to its category membership. Proximity of the points represents more similar brain activation patterns for the stimuli. Note that in the FFA and OFA, the illusory faces are more separated from the matched objects and closer to the human faces compared to LO and PPA.

**Fig. 3:**
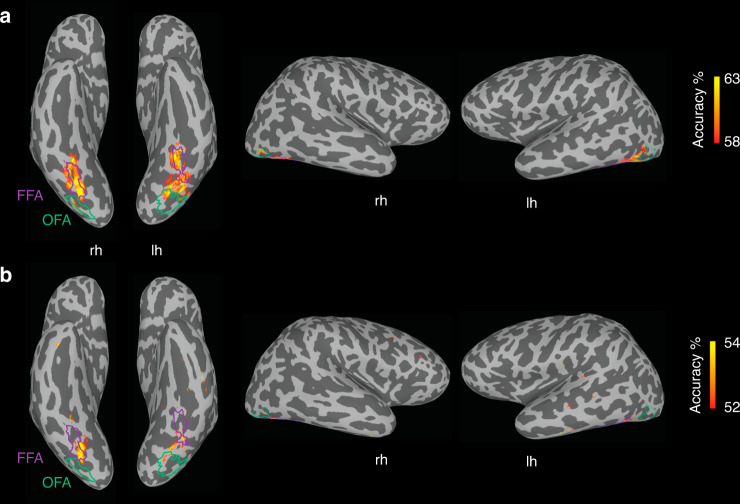
Localized sensitivity to illusory faces. fMRI cross-decoding searchlight results for **a** human faces vs. objects and **b** illusory faces vs. objects. For both comparisons, the location of greatest decoding accuracy is within ventral temporal cortex and overlaps with FFA particularly in the right hemisphere. The location of greatest cross-decoding for illusory faces vs. matched objects (across exemplars) is a subset of the area for human faces vs. objects. For illustration, the approximate boundaries of functionally defined FFA (purple) and OFA (green) as defined by the location of overlapping surface nodes across individual participants (node inclusion threshold = 3/16 participants for OFA, 4/16 participants for FFA) are drawn on this example inflated surface for comparison with the searchlight results.

Our first aim was to characterize which of the category-selective regions in higher-level visual cortex are sensitive to the presence of an illusory face in an inanimate object. In each participant, we identified the face-selective fusiform face area (FFA) and occipital face area (OFA), an object-selective lateral occipital (LO) area, and the scene-selective parahippocampal place area (PPA) based on independent functional localizer runs (Fig. 2a). We used a cross-decoding analysis to test which brain regions had activity that could distinguish between the three categories of experimental stimuli (Fig. 1d). A linear support vector machine was trained to classify which category of stimulus a subject was viewing based on the patterns of BOLD activation across voxels in a given region of interest. The classifier’s performance was tested on data from stimulus exemplars of each category not used in the training set, thus correct classification required generalization to new stimuli, ruling out an explanation based on visual features associated with specific images in the training set. Statistical significance was assessed using one-sample *t*-tests (one-tailed) against chance decoding performance (50%). The FDR adjustment was made to all reported *p* values to control for multiple comparisons. In all four regions, human faces could be distinguished from objects with an illusory face (FFA: *t*_(15)_ = 6.33, *p* = 0.00004, *d* = 1.58, 95% CI: 58.83, Inf; OFA: *t*_(15)_ = 4.37, *p* = 0.0005, *d* = 1.09, 95% CI: 54.20, Inf; LO: *t*_(15)_ = 5.20, *p* = 0.0002, *d* = 1.30, 95% CI: 56.04, Inf; PPA: *t*_(15)_ = 4.08, *p* = 0.0008, *d* = 1.26, 95% CI: 52.04, Inf) or without an illusory face (FFA: *t*_(15)_ = 7.48, *p* = 0.00001, *d* = 1.87, 95% CI: 62.53, Inf; OFA: *t*_(15)_ = 5.13, *p* = 0.0002, *d* = 1.28, 95% CI: 57.15, Inf; LO: *t*_(15)_ = 4.68, *p* = 0.0003, *d* = 1.17, 95% CI: 56.83, Inf; PPA: *t*_(15)_ = 5.04, *p* = 0.0002, *d* = 1.02, CI: 52.84, Inf) (Fig. 2b). However, illusory faces could only be discriminated from similar matched nonface objects in face-selective FFA (*t*_(15)_ = 2.54, *p* = 0.015, *d* = 0.64, 95% CI: 50.64, Inf) and OFA (*t*_(15)_ = 2.34, *p* = 0.020, *d* = 0.59, 95% CI: 50.60, Inf), but not in object-selective LO (*t*_(15)_ = 1.35, *p* = 0.11, *d* = 0.34, 95% CI: 49.71, Inf) or scene-selective PPA (*t*_(15)_ = 0.27, *p* = 0.40, *d* = 0.07, 95% CI: 48.92, Inf). Overall, these results show that the perception of an illusory face in an object modulates the brain’s response to that object in face-selective cortex only. The univariate results are also consistent with the role of face-selective FFA and OFA in distinguishing illusory faces from matched objects (Supplementary Fig. 2). These results were further supported by a whole-brain decoding searchlight (Fig. 3), which showed that the main areas of successful decoding of human faces from objects (Fig. 3a) and illusory faces from objects (Fig. 3b) align with functionally defined FFA and OFA, with some additional area located on the ventral surface between these ROIs.

To further characterize the brain’s response to illusory faces, we applied representational similarity analysis (RSA)^18,19^. We constructed dissimilarity matrices by correlating the patterns of fMRI BOLD activation between each pair of stimuli and taking 1-correlation to convert to a measure of pattern dissimilarity (Fig. 2c). For visualization, we applied multidimensional scaling (MDS) to the resulting matrices for each region of interest and plotted the first two dimensions (Fig. 2d). Note that in FFA and OFA, human faces are represented similarly to each other, and illusory faces are more similar to human faces than matched objects are (see blue regions in Fig. 2c). In comparison, in LO and PPA, all objects are grouped together regardless of whether they contain an illusory face or not. This grouping is consistent with the lack of illusory face cross-decoding in these regions. Overall, this analysis demonstrates that illusory faces are represented uniquely compared to both human faces and nonface objects in face-selective cortex. In contrast, illusory faces are not distinct from ordinary objects in either object or scene-selective cortex.

### Illusory faces are rapidly decoded from whole-brain activity

**Fig. 4:**
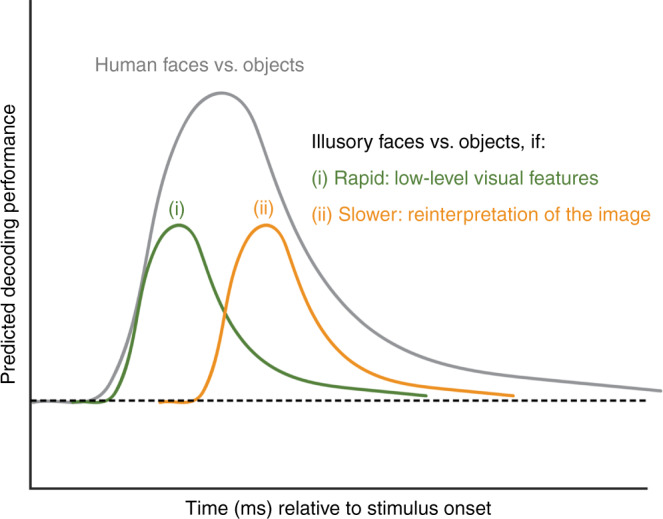
Schematic diagram showing predicted results for MEG cross-decoding of illusory faces vs. objects based on two possible accounts: (i) rapid processing based on low-level visual features (green line), or (ii), slower reinterpretation of the image (orange line). Relative to decoding human faces vs. objects (gray line), performance is expected to be reduced because illusory face images share many more visual features with the matched object images than they do with human face images, thus brain activation patterns for these categories are expected to be less separable. See Fig. 5a for the empirical results.

One of our primary goals was to determine whether illusory faces are processed rapidly based on activation of a broadly tuned face-detection mechanism by their low-level visual features, or alternatively, whether they result from a slower cognitive process requiring the reinterpretation of the image. Individual stimuli were readily decodable from the MEG whole-brain activation patterns after stimulus onset with similar decoding within each category (Supplementary Fig. 3). To address the processing of illusory faces, we focused on the category level and evaluated how quickly illusory faces could be distinguished from both human faces and matched objects from time-varying whole-brain activity measured with MEG. We used a cross-decoding approach (training and testing on different exemplars), so that successful classification required the classifier to generalize to new stimuli. We generated predictions for the cross-decoding results based on the two possible accounts (Fig. 4). If illusory faces activate a rapid neural pathway based on low-level visual features, we would expect peak decoding at a similar time to that for human faces (green line, Fig. 4). Alternatively, if illusory face perception requires a slower cognitive process based on reinterpreting the image, peak decoding should occur later relative to stimulus onset (orange line, Fig. 4). In both cases, we would expect reduced performance for cross-decoding illusory faces from matched objects compared to decoding real faces vs. objects because the illusory faces share many more visual and semantic features with the objects; and consequently their brain activation patterns will be more similar, making the classification problem more challenging.

To reveal at what time the presence of an illusory face in an object could be decoded from the brain activation patterns, we trained a separate binary LDA classifier to discriminate between each of the three pairs of our three stimulus categories (i.e., human faces vs. objects, illusory faces vs. objects, and illusory faces vs. human faces). Importantly, here we used a cross-decoding approach, training the classifier on brain activation patterns in response to a subset of the 96 stimuli, and testing the classifier’s performance on generalizing to brain activation patterns elicited by a new subset of stimuli not used in training (Fig. 1d). This means that successful classifier performance in discriminating each of the three stimulus categories (human faces, illusory faces, nonface objects) from the time-varying activity across MEG sensors could not be explained by sensitivity of the classifier to brain signatures associated with the visual properties of particular images, since successful performance requires generalizing to new examples of each category.

**Fig. 5:**
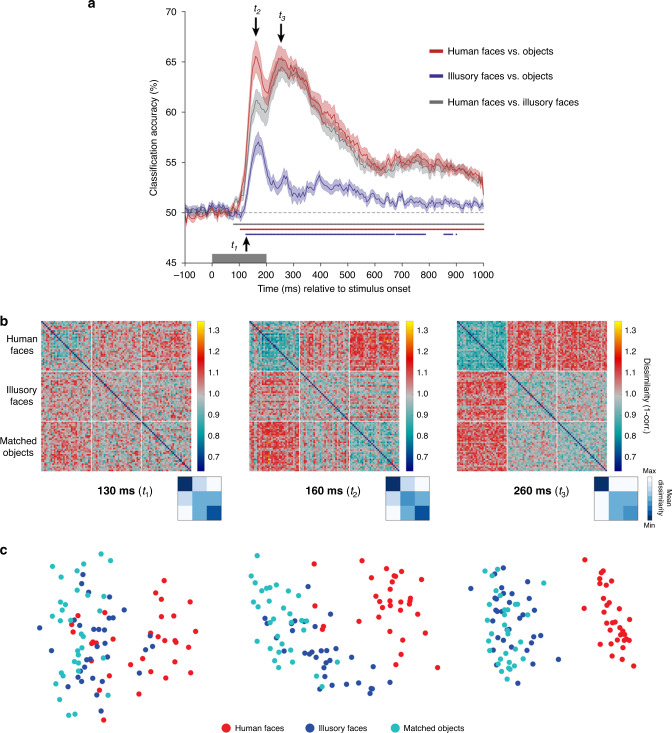
MEG results showing a rapid transformation in the representation of illusory faces over time. **a** Cross-decoding results across time for all three category comparisons. Mean classifier performance is plotted across time relative to stimulus onset, averaged over *N* = 22 participants. Shaded area represents SEM. Multiple comparisons were controlled for using Threshold-Free Cluster Enhancement as implemented in CoSMoMVPA^26^. Stimulus duration is indicated by the gray bar from 0–200 ms on the *x*-axis. Chance performance is 50%, indicated by the dashed line. Colored disks along the *x*-axis indicate statistically significant timepoints. By 130 ms (*t*_1_) from stimulus onset, all three comparisons can be significantly decoded. There is an initial peak at ~160 ms (*t*_2_) for all comparisons, and a second peak ~260 ms (*t*_3_) for decoding human faces from all objects with or without a face, that is absent for decoding illusory faces from matched objects. These three timepoints of interest are used as the focus for the subsequent analyses. Source data are provided as a Source data file. **b** Representational dissimilarly matrices (96 × 96) for all stimuli for the three timepoints of interest (130, 160, and 260 ms post stimulus onset). The dissimilarity is calculated by taking 1-correlation (Spearman) between the MEG activation patterns for each pair of stimuli. The colorbar range is scaled to the max and min of the dissimilarity values across all timepoints for visualization (see Supplementary Movie 1 for complete time-varying RDM). White lines indicate stimulus category boundaries. Insets show 3 × 3 matrices for each time point averaged by category, excluding the diagonal. Source data are provided as a Source data file. **c** Visualization of the dissimilarity matrices in (**b**) using multidimensional scaling. The first two dimensions following MDS are plotted. Each of the points representing the 96 stimuli is colored according to its category membership. Proximity of the points represents more similar brain activation patterns for the associated stimuli.

All three categories could be decoded from each other within the 200 ms stimulus presentation duration (Fig. 5a). As outlined in Fig. 4, we were interested in the relative time course of decoding for illusory faces vs. objects in comparison to that for human faces vs. objects. Human faces could be discriminated from nonface objects soon after stimulus onset (red line), with two peaks in classifier performance at ~160 and ~260 ms. Importantly, decoding of illusory faces from objects also peaked at ~160 ms (blue line). This is consistent with rapid processing of illusory faces based on visual features, instead of a slower cognitive reinterpretation of the image (compare to predictions in Fig. 4). A result we did not anticipate was the more transient nature of the result for decoding illusory faces from objects compared to real faces. Notably the second peak at ~260 ms observed for human faces is absent for illusory faces, and although decoding remains significant after onset for most of the 1100 ms analysis window, its magnitude is vastly reduced relative to that for real faces. We investigate this further using RSA to probe the brain’s representation of illusory faces in more detail.

### An early face response followed by rapid reorganization

The decoding results are indicative of a dynamic change in how illusory faces are represented relative to real faces and similar nonface objects over a relatively brief time period of ~200 ms. To investigate this further, we applied RSA^18,19^. For each pair of 96 stimuli, we correlated their time-varying MEG activation patterns across all sensors at each time point. This produced a representational dissimilarity matrix (RDM, Fig. 5b, Supplementary Movie 1), which depicts the relative similarity between the brain’s representation of each pair of stimuli (1-Spearman’s *R*). Here we focus on three timepoints of interest based on the cross-decoding results in Fig. 4a: *t*_1_ (130 ms) corresponds to the first time point after all three category comparisons were significant, *t*_2_ (160 ms) is when the first decoding peak occurs for all three category pairs, and *t*_3_ (260 ms) is the time of the second decoding peak for decoding human faces from all objects with or without a face, which is notably absent for decoding illusory faces from nonface objects. We additionally used MDS to produce a visualization of the dissimilarity matrices, plotting the first two dimensions (Fig. 5c, Supplementary Movie 2). Each of the points representing the 96 stimuli is colored according to its category membership. Closer proximity of the points represents more similar brain activation patterns for the associated stimuli.

The most striking feature of the representational structure is how it changes dynamically across these three timepoints, as visualized in the dissimilarity matrices (Fig. 5b) and MDS plots (Fig. 5c). At 130 ms the human faces are already more similar to each other than to illusory faces or objects (Fig. 5b), as shown by the clustering of the human face exemplars in the MDS plot (Fig. 5c). Notably, a few illusory face exemplars are clustered with the human faces (Fig. 5c). By 160 ms, the illusory faces have started to segregate from the matched objects (Fig. 5c), and have become more similar to the human faces than the matched objects are (Fig. 5b). There is a categorical shift in the representational organization between 160 and 260 ms: initially the illusory faces are distinct from the matched objects, but only 100 ms later, the illusory faces are grouped with the matched objects (Fig. 5c). As with the rapid onset of category-level cross-decoding (Fig. 5a), this shows that illusory faces are rapidly processed. These errors of face detection are initially treated more like real faces than matched objects are, however, the human brain rapidly resolves this detection error and they are represented more similarly to objects in less than a quarter of a second.

### The contribution of visual features to rapid face detection

The decoding and RSA results show an early response to illusory faces in the patterns of brain activity measured with MEG. This is consistent with engagement of a rapid process for face detection (see Fig. 4) rather than a slower cognitive reinterpretation of the image. Presumably a fast face-detection process would be based on simple or coarse visual features associated with real faces. If this is the case, we might expect that the early stages of the MEG response to illusory faces would be driven by simple or coarse visual features in the images. Here we aim to examine the extent to which two computational models of visual features can explain the brain’s response to illusory faces by comparing their representation of the stimuli to that measured by behavioral ratings of “face-ness” and the brain activation patterns measured by MEG. We selected two well-established computational models that emphasize different visual features in an image: Graph-Based Visual Saliency^20^ (GBVS) and the GIST^21^ visual feature model. Prior research has suggested that features important for face recognition typically consist of high contrast regions^22–24^. Computational models of visual saliency such as GBVS aim to predict where a human observer would look in an image. Given that regions of high saliency frequently correspond to areas of high contrast, we reasoned that this class of model may approximate the type of visual characteristics relevant to the face-detection mechanism. In contrast, the GIST^21^ visual feature model is based upon characterizing the spatial distribution of content across an image, and may be able to capture different low-level visual features associated with illusory faces.

**Fig. 6:**
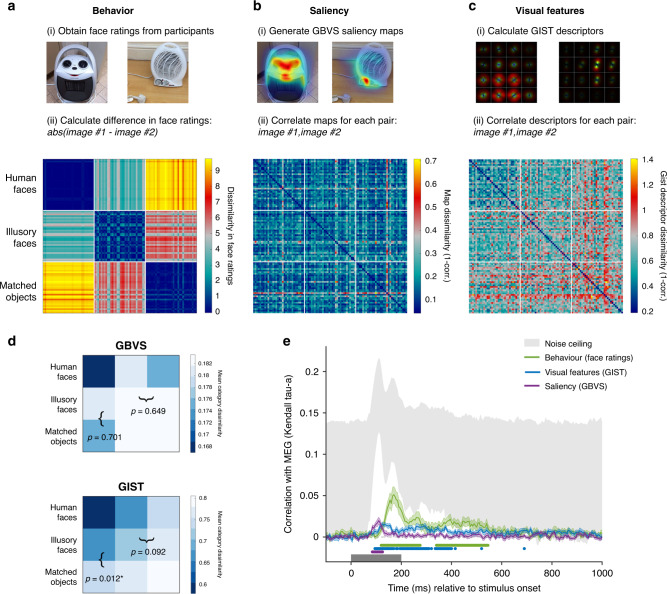
Linking MEG representations to models of visual saliency, visual features, and behavior. Construction of representational dissimilarity matrices for comparison with the MEG data for **a** visual saliency based on the GBVS model, **b** visual features based on the GIST model, and **c** behavior based on participants’ face ratings. Source data are provided as a Source data file. **d** Category-averaged RDMs were constructed for the saliency and visual feature models by averaging the mean dissimilarities for each exemplar in (**a**, **b**) respectively to produce 3 × 3 matrices. We tested (i) whether illusory faces were more similar to human faces than matched objects were and (ii) whether illusory faces were more similar to each other on average than to matched objects by subtracting the relevant squares in the category-averaged RDMs (marked with brackets). Reported *p* values are FDR corrected values (to control for multiple comparisons) from a two-sided permutation test (1000 permutations of the category labels of the original 96 × 96 RDM). Asterisks (*) indicate statistical significance at *p* < 0.05. **e** Correlation between the behavioral, saliency, and visual feature models in (**a**–**c**) with the time-varying MEG dissimilarity matrix. Shaded area represents SEM. The noise ceiling marked in gray represents the estimate of the maximum correlation possible given the data^25^. Statistically significant timepoints are indicated by colored disks along the *x*-axis. Multiple comparisons were controlled for using Threshold-Free Cluster Enhancement as implemented in CoSMoMVPA^26^. The saliency model significantly correlates with the MEG data from 85–125 ms post stimulus onset. The main significant correlation between the visual feature model and the MEG data occurs from 95–400 ms, while the behavioral model correlates in two time-windows, from 120–275 and 340–545 ms. Source data are provided as a Source data file.

Before testing the correlation with the MEG data, we examined the representational dissimilarity matrices (RDMs). First, we constructed a dissimilarity matrix based on the behavioral ratings of “face-ness” for the stimuli (Fig. 6a). Notably, the behavioral matrix reveals that illusory faces are more similar to human faces than the matched objects are. Further, different illusory face exemplars are also more similar to each other than they are to the matched objects. These relationships are expected from the behavioral results showing that illusory faces were rated as more face-like than matched objects, but less so than human faces (Fig. 1b). Next, we built separate dissimilarity matrices for the two models by correlating the saliency maps generated from the GBVS model (Fig. 6b) and the gist descriptors produced by the GIST model (Fig. 6c) for each pair of images. To test whether the representation of illusory faces generated from the GBVS and GIST models is distinct from that of human faces and matched objects we averaged the dissimilarity across all 32 exemplars of each category, producing 3 × 3 category dissimilarity matrices (Fig. 6d). For the GIST model, the category-averaged RDM revealed that illusory faces were on average more similar to human faces than the matched objects (mean difference = −0.0696, *p* = 0.012, permutation test with FDR corrected *p* values). This was not the case for the GBVS saliency model (mean difference = 0.0063, *p* = 0.701). However, illusory faces were not more similar to each other on average than they were to matched objects for either the GBVS (mean difference = −0.0000004, *p* = 0.649) or GIST (mean difference = −0.0450, *p* = 0.092) models. Overall, these results suggest the GIST visual feature model is sensitive to some of the distinguishing features of illusory faces, however, neither the GIST or GBVS models capture the clear perceptual differences revealed by the behavioral ratings of how easily a face can be perceived in each image (Fig. 6c).

To examine to what degree these model representations explain the time-varying representation of the stimuli measured with MEG, we correlated each of the three model RDMs (Fig. 6a–c: behavior, saliency, and visual features) with the time-varying MEG RDM (Supplementary Movie 1). Importantly, we removed the human faces from both model and MEG RDMs for this analysis. A preliminary analysis showed that human faces produce a strong neural response, which dominated the results, and consequently only confirmed that human faces were represented differently than the other stimuli. As our focus here is on how illusory faces modulate the representation of an object, including human faces would inflate any observed correlations between the brain representations and the models. The saliency RDM significantly correlated with the MEG RDM from 85–125 ms after stimulus onset (Fig. 6f), with one discreet peak at 110 ms. In comparison, a more sustained correlation was observed with the visual feature RDM, starting at 95 ms after stimulus onset and continuing throughout the stimulus presentation. Consistent with the observed differences in the category-averaged RDMs (Fig. 6d), this difference in the time course of the correlation with the MEG RDMs suggests that the saliency and visual feature models pick up on different aspects of the stimuli. The behavioral RDM based on the face ratings had a sustained correlation with the MEG RDM from 120 ms, with a peak at 170 ms (Fig. 6b). The correlation with behavior also reached much closer to the noise ceiling, an estimate of the maximum correlation expected with the MEG data^25^. The peak correlation with behavior occurred around the time of the first decoding peak at ~160 ms (Fig. 5a), which is notably later than the correlation with the saliency or visual feature models. The finding that the correlation with behavior was stronger overall than for either computational model suggests that these models capture an initial stage of processing, but not the full time course.

### An early face-like response in FFA

**Fig. 7:**
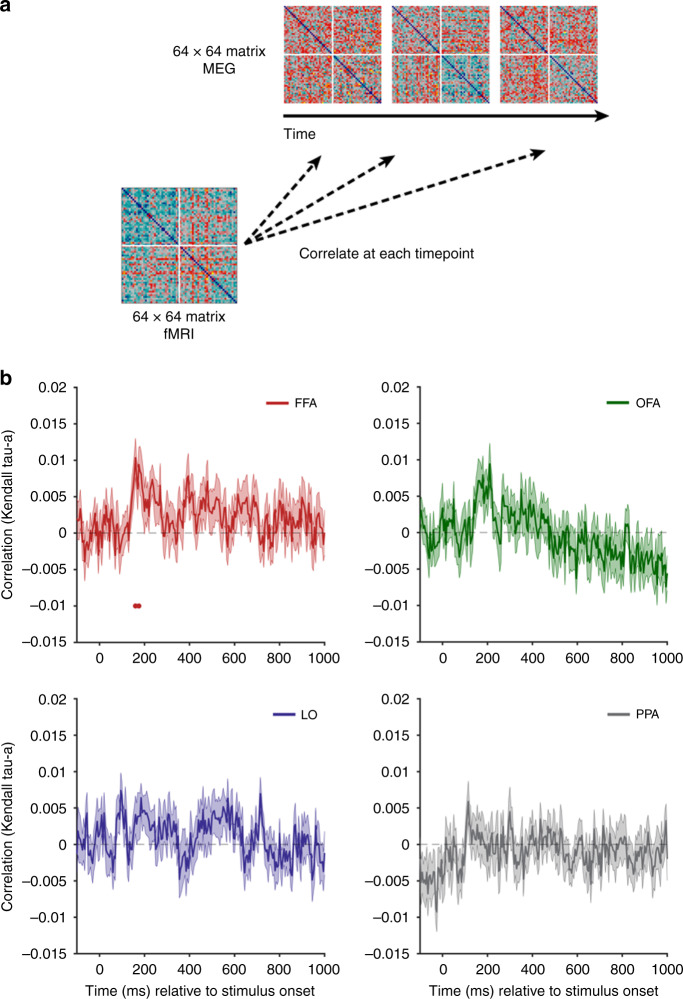
fMRI-MEG fusion. **a** The fMRI dissimilarity matrices (1-correlation) for each of the four ROIs were correlated with the time-varying MEG dissimilarity matrix (1-correlation). Note that real human faces (*n* = 32) were removed from this analysis so the matrices are 64 × 64. **b** Results of fMRI-MEG fusion. The mean correlation between each of the RDMs for the four ROIs with the time-varying MEG RDM is plotted relative to stimulus onset, averaged over *N* = 22 participants. Statistical significance is indicated by colored disks along the *x*-axis. Error is SEM. Source data are provided as a Source data file.

In order to link the MEG and fMRI data, we used a fusion approach^27^. We correlated the fMRI RDMs for each of the four regions of interest with the time-varying MEG RDM at every time point (Fig. 7a). As for the RSA model comparison with saliency and behavior, we removed human faces from this analysis. This makes the analysis more conservative, and any significant correlations are reflective of the representation of objects with and without an illusory face. The representation in the FFA significantly correlated with the MEG data for a brief period post stimulus onset (160, 175 ms). The correlation was not significant at any time for OFA, LO, or PPA. This suggests that the face response to illusory faces demonstrated by the peak correlation with behavior at 170 ms (Fig. 6b) and in cross-decoding at 160 ms (Fig. 5a) in the MEG data has a likely origin in FFA.

## Discussion

Our results reveal the representation of natural errors of face detection in the human brain. With fMRI and multivariate pattern classification methods, we found that a face-like response to illusory faces was restricted to face-selective regions in occipital–temporal cortex. In scene and object areas, illusory faces were instead represented more similarly to objects. Exploiting the much higher temporal resolution of MEG, we showed that illusory faces are initially represented as more face-like in their brain activation patterns than similar matched objects. However, only ~100 ms later, illusory faces are represented as more object-like, consistent with a rapid resolution of the detection error. Thus, although illusory faces in objects are perceived as having a persistent dual identity (face, object), their neural representation quickly shifts in its relative weighting of these two identities over time. This is consistent with the rapid activation of a broadly tuned face-detection system, which is tolerant of substantial visual variance in the definition of facial features. Following the initial face-detection process, which possibly occurs via a subcortical route^28^, the subsequent resolution of the error is likely driven by processing in cortical areas involved in face recognition^1,4,5^.

It is currently debated to what degree representations in ventral temporal cortex are driven by the appearance or visual properties of an object vs. its semantic meaning^29–34^. Recently it was demonstrated that in ventral occipitotemporal cortex, objects that look like another object (for example, a cow-shaped mug) have BOLD activation patterns that are more similar to what object they look like (e.g., a cow) than to their actual object identity (e.g., a mug)^30^. Similarly, it has been debated whether object representations in ventral temporal cortex are organized by an overall semantic principle such as animacy^14^ or real-world size^35,36^, or by visual properties that co-vary with these categories^29,31,32,34^. Our data offer interesting insights into this issue. Despite sharing more visual properties and their semantic identity with the yoked objects, the illusory faces were represented as more similar to human faces than the yoked objects were in face-selective FFA and OFA, and for a brief transient period in the first quarter of a second as revealed by whole-brain MEG. This suggests that the response to stimuli in which meaning and visual appearance are dissociated is not homogenous throughout ventral visual cortex, and further, that these representations exhibit temporal variability rather than stability over time in the brain’s response.

Our results are able to adjudicate between two alternative mechanisms for illusory face perception, providing evidence for a rapid detection mechanism rather than a slower cognitive reinterpretation of the object as a face. The early timing of the correlation between the MEG data and the saliency and visual feature models relative to that with behavior suggests that illusory face perception is at least partially driven by particular low or mid-level features. However, neither computational model fully explains the behavioral or brain data. The differences in timing reflected by the transient correlation with the GBVS saliency model and the more sustained correlation with the GIST visual feature model suggest that the two models isolate different visual characteristics that might drive the erroneous face-detection response to illusory faces at different stages of processing. Previous fMRI investigation of face detection in visual noise has suggested the eye and mouth regions are important features in modulating the response of face-selective visual areas, consistent with the idea of a simple template for face detection^37^. A rapid subcortical route for face detection has been proposed based on multiple lines of evidence^28^, involving the superior colliculus, pulvinar, and amygdala. The amygdala has been implicated in orienting toward faces in primates, and amygdala lesions impair this tendency for both real and illusory faces^3^. Along with the timescale of the early confusion we observe between real and illusory face processing, this is suggestive of a possible role for the amygdala in falsely detecting faces in everyday objects.

Beyond revealing a rapid neural mechanism underlying illusory face perception, our data offer insight into the distinction between face detection and face recognition in the visual system. Visual detection and recognition have competing requirements, which must be adequately weighted in a biological system in order to optimally direct the behavior of the organism. To improve detection a system needs to maximize sensitivity, yet recognizing an object as belonging to one category or another requires precise tuning to visual features. Neurons in face-selective cortical areas are tightly tuned to particular features associated with real faces^6,11,38^, and this tight tuning might be expected to result in poor detection. In contrast, the existence of face pareidolia in a wide range of objects is suggestive of a broadly tuned face-detection system. Our results suggest that this broadly tuned face detector results in compelling errors, yet these errors are quickly resolved, likely via a rapid subcortical route for face detection. The role of individual face-selective regions in the human brain is still being revealed^1^. If rapid face detection is served by a subcortical route, processing in cortical areas may be focused on different aspects of the complex task of face recognition. This functional anatomical distinction may explain how the human visual system balances the competing requirements of face detection vs. recognition.

Although we observe a rapid face-like response to illusory faces in objects, there are substantial differences in the representation of human faces vs. illusory faces. The fMRI data showed that in face-selective FFA and OFA, human faces have more similar within-category activation patterns than illusory faces, which are less similar to each other. Further, in nonface areas such as LO and PPA, the illusory faces group with objects rather than human faces. This is similar in the time domain revealed by MEG, in which illusory faces have a more transient face-like response than human faces, and are much more similar to nonface objects within a couple 100 ms. The fact that human faces and illusory faces are readily decoded from each other in both fMRI and MEG is also indicative of differences in their representation. Overall, this difference is consistent with the interpretation of illusory faces as a quickly resolved error of a broadly tuned face-detection system^2,3^. Thus although illusory faces elicit a face-like response, they are not represented in the same way as human faces within the visual system.

Our unexpected finding that the face-like response to illusory faces is not only rapid, but is also brief, underscores the importance of investigating temporal dynamics in understanding the neural mechanisms of visual perception. Our results are consistent with prior MEG results suggesting differential stages of information processing of faces that unfold over a few 100 ms^39^. Recently, an MEG study using famous faces reported that certain features such as gender and age were evident in the whole-brain representation of the face before identity^40^. Similarly, neurons in macaque temporal cortex are known to respond to global information about faces in the earliest part of the response, with finer-scale information about identity or expression emerging later^41^. Broadly consistent with our finding of a rapid face-like response to illusory faces, an EEG study reported an increased neuronal response across frontal and LO cortex within 500 ms to white noise patterns in which observers mistakenly thought they saw a face^42^. Our result reveals a transformation of the representational space that occurs in a fraction of a second. Together these results are indicative of the type of insights into neural mechanisms that are inaccessible at the slower temporal resolution of the fMRI BOLD response^43^. A curious feature of illusory faces is that the percept of a face persists well beyond the initial few 100 ms in which we now know they are represented in the brain as more face-like. This suggests that the MEG data are reflective of the initial processing that leads to the misperception of a face in these objects.

In sum, illusory faces are represented uniquely in the brain compared to both real human faces and similar objects that do not have illusory facial features. The presence of an illusory face initially results in a rapid face-like response to an inanimate object, possibly via a subcortical route. However, this detection error is rapidly resolved and the brain’s representation transforms such that illusory faces are represented more similarly to matched objects than human faces within a couple of 100 ms. This result underscores the importance of considering temporal dynamics in understanding human cognition.

## Methods

### Participants

All imaging experiments were approved by the Human Research Ethics Committee of Macquarie University and participants received financial compensation for their time. The online experiments were conducted on Amazon Mechanical Turk following guidelines set by the NIH Office of Human Subjects Research Protections, and participants were also compensated for their time. All experiments were conducted in accordance with the Declaration of Helsinki and informed consent was obtained from each participant. In total, 22 participants (8 male, 14 female, mean age 26.2 years, range 18–41 years) completed the MEG experiment. In total, 21 participants (11 male, 10 female, mean age 25.4 years, range 20–36 years) participated in the fMRI experiment. *N* = 4 participants were removed from the fMRI analysis due to inability to define category-selective regions from their localizer data and *N* = 1 was excluded due to failing to complete the experiment, leaving *N* = 16 fMRI datasets for analysis. In total, 20 participants (12 female, 7 male, 1 other) completed the behavioral experiment online via Amazon Mechanical Turk. Note that the human face images used in the experiments are not shown in the paper because we do not have the rights to publish them. The original face stimuli used in the experiments are available at the Open Science Framework website for this project: https://osf.io/9g4rz. The human faces shown in this figure are similar photographs taken of lab members who gave permission to publish their identifiable images.

### Visual stimuli

The stimulus set consisted of 96 photographs sourced from the internet (32 illusory faces, 32 matched nonface objects, 32 human faces). For each of the 32 examples of illusory faces in inanimate objects (e.g., bell peppers, backpack, coffee cup), we selected a matched object of the same type and as visually similar as possible, but which did not contain an illusory face (Fig. 1a). The illusory face images have a high degree of variance in the visual appearance of their illusory facial features, compared to human facial features. In addition, the illusory face images have greater variance in visual properties such as color, orientation, and face size, compared to the controlled face images typically used in experiments on face processing. For these reasons, the set of 32 human faces was selected to have a high degree of variance across age, gender, race, facial expression, and head orientation in order to match the high variance of the illusory face image set. Human faces were not individually matched to the illusory faces because of the ambiguity in defining parameters such as age, race and gender for illusory faces. However, we did match the number of images containing more than one face across human and illusory face sets. Two of the illusory face images contained two faces, so we included two images in the human face set that also had two faces. All images were cropped to a square image and resized to 400 × 400 pixels, but no other manipulations were made.

### fMRI data acquisition

The fMRI experiment consisted of a structural anatomical scan, two functional localizer runs, and seven experimental runs. MRI data were acquired with a 3T Siemens Verio MRI scanner and a 32-channel head coil at Macquarie Medical Imaging, Macquarie University Hospital (Sydney, Australia). A high-resolution T1-weighted structural MRI scan (3D-MPRAGE sequence, 1 × 1 × 1 mm voxel size, in-plane matrix size: 256 × 256, 176 slices, TR = 2 s, TE = 2.36 ms, FA = 9°) was collected for each participant at the start of the session. Functional scans were acquired with a 2D T2*-weighted EPI acquisition sequence: TR = 2.5 s, TE = 32 ms, FA = 80°, voxel size: 2.8 × 2.8 × 2.8 mm, in-plane matrix size: 92 × 92. A whole-brain volume containing 42 slices was collected. In total, 128 volumes were collected per localizer run (5 min each), and 218 per experimental run (9 min each). One localizer run was collected immediately after the structural scan, followed by the seven experimental runs, and then the second localizer run. In total the scanning session took <90 min.

Stimulus presentation scripts were written in MATLAB using functions from the Psychtoolbox^44–46^ and run on an Apple MacBook Pro with Mac OSX. Visual stimuli were displayed using a flat-panel MRI-compatible 32″ Cambridge Research Systems BOLDscreen with resolution 1920 × 1080 and viewing distance 112 cm. Experimental stimuli subtended 7.4 × 7.4° and localizer stimuli 8.8 × 8.8°. Behavioral responses were collected using a Lumina MRI-compatible button box.

*fMRI category-selective localizer*: two independent localizer runs were used to define the category-selective regions FFA, OFA, LO, and PPA individually in each participant. The functional localizer stimuli were color pictures of faces, places, objects, and scrambled objects (480 × 480 pixels). In total, 54 images for each category were selected from The Center for Vital Longevity Face Database^47^, the SUN397 database^48^, and the BOSS database^49,50^ respectively. Scrambled objects for localizing object-selective region LO^51^ were pregenerated in MATLAB by randomly scrambling each object image in an 8 × 8 grid and saving the resulting image. Localizer runs began with a 4 s fixation period before the first stimulus block. For each stimulus class, there were 3 unique blocks of 18 images. Participants performed a 1-back task, pressing a key each time an image was repeated twice in a row. Every time a block was run, the images were presented in a random order and two random images were repeated twice for the 1-back task. Each of the 20 images within a block (18 unique + 2 repeats) was shown for 600 ms followed by a 200 ms interstimulus interval. The 16 s stimulus blocks alternated with 10 s fixation blocks. Each of the four stimulus categories was repeated three times per 5-min localizer run, once per unique image set. The order of block presentation was in a pseudorandom order, with two different orders counterbalanced across runs for each participant.

*fMRI experimental runs*: the fMRI experiment used a rapid event-related design^14^, in order to measure the BOLD activation patterns for each individual stimulus. Each of the 96 stimuli was shown once per run, in random order. In each 4 s trial, stimuli were presented for 300 ms at the start of the trial on a gray background. For the remaining time in each trial after stimulus offset, a gray background with a fixation cross was shown. In addition to the 96 stimulus trials, 32 blank null trials (4 s duration each) were inserted randomly in the sequence for each run and each participant. Finally, four null trials were inserted at the start and end of each run. This produced 136 trials total per run for a run duration of 9 min. Each participant completed seven experimental runs.

A task unrelated to face detection was used in order to maintain participants’ alertness during the fMRI experiment. Each image was presented tilted slightly by 3° to the left or right of center (Fig. 1c) and participants’ task was to report the direction of tilt with a keypress using a Lumina MRI-compatible button response pad. At the end of each run, participants received on-screen feedback on their performance on the rotation task (accuracy, reaction time, number of missed trials). For each run and participant, the tilt of each stimulus was pseudo-randomly allocated such that tilt direction was counterbalanced equally within each of the three stimulus categories (faces, illusory faces, and matched objects). Averaged across participants and runs, the mean task accuracy was 92.5% (SD = 8.6%). Eye movements were not recorded, however, the brief stimulus duration prevented any substantial eye movements during stimulus presentation.

*fMRI preprocessing*: fMRI data were preprocessed using the AFNI^52^ software package. EPIs were slice-time corrected, motion-corrected, and co-registered to the participant’s individual anatomical volume. Spatial smoothing of 4 mm full-width at half-maximum was applied to the localizer runs only, no smoothing was conducted on the experimental runs. All analyses were conducted in the native brain space of each participant.

### Functional ROI definition

Four functional ROIs were defined in each participant’s brain from the independent localizer data: FFA, OFA, LO, and PPA. Data from the two independent localizer runs were entered as factors into a GLM in AFNI^52^ to estimate the beta weights for faces, scenes, objects, and scrambled objects. Contrasts of faces–scenes, scenes–faces, and objects–scrambled objects produced t-maps used to define the boundaries of each ROI. Cortical reconstruction was performed using Freesurfer 6.0 from the structural scan for each participant. Inflated surfaces were visualized in SUMA^53^ for functional ROI definition, with the results of the GLM overlaid as t-maps. FFA and OFA were defined as the contiguous cluster of voxels in the fusiform gyrus and LO area, respectively, produced from the contrast between faces vs. scenes. LO was defined as activation on the LO surface from the contrast between objects and scrambled objects. Finally, PPA was defined as the peak cluster of activation in the parahippocampal gyrus produced by the contrast between scenes vs. faces. To ensure unique ROIs, any overlapping voxels in the four regions were preferentially allocated to the face-selective regions, and any voxels allocated to both FFA and OFA were removed from both ROIs. The mean size of each ROI averaged across participants was 271 voxels for FFA (range: 86–577), 190 voxels for OFA (range: 101–316), 337 voxels for LO (range: 70–543), and 393 voxels for PPA (range: 277–712).

### fMRI multivariate pattern analysis

Decoding analysis was performed using The Decoding Toolbox (TDT)^54^ and MATLAB. Decoding was performed with a linear SVM in each ROI on the beta weights estimated in a GLM using AFNI^52^ for each of the 96 stimuli, producing a separate beta weight for each run (i.e., 7 beta weights per stimulus). For cross-validation we used a combined leave-one-run-out and leave-one-exemplar-out procedure as implemented in TDT^54^. Cross-decoding for each category pair (faces vs. objects, faces vs. illusory faces, illusory faces vs. objects) was performed by training the classifier on the data for all exemplars except one pair (*N*−1 = 31), which served as the test set. Leave-exemplar-out cross-validation was performed by repeating this process iteratively such that each exemplar was in the test set once. Classifier accuracy was averaged across all cross-validation folds for each ROI and category pair (Fig. 2b). Statistical significance was assessed using one-tailed t-tests with control for multiple comparisons (at *α* < 0.05) implemented using the FDR procedure for adjusting *p* values described by Benjamini and Hochberg^55^ and implemented with MATLAB’s *mafdr* function.

RSA was conducted using MATLAB. RDMs for each ROI and participant were constructed by correlating the beta coefficients for each pair of stimuli across voxels in the ROI, and taking 1-correlation (Spearman’s rho) to convert to a dissimilarity measure^18,19^. The mean of individual participants’ RDMs produced one RDM per ROI (Fig. 2c). MDS was performed using the MATLAB function *cmdscale* on the average RDM across participants for each ROI, with the first two dimensions plotted for visualization (Fig. 2d).

A whole-brain decoding searchlight was conducted using the Newton linear SVM classifier implemented in TDT^54^ with a searchlight radius of 3 voxels. We used a leave-exemplar-out cross-validation approach as implemented for the ROI decoding analysis, however, to improve performance for the searchlight we used the faster Newton implementation of SVM and did not implement leave-one-run-out cross-validation in addition to leave-exemplar-out. The searchlight was conducted in each participant’s native brain space, and then the results were mapped on to surface nodes in SUMA^53^ for visualization of the average group decoding maps for all participants (Fig. 3).

### MEG data acquisition

MEG data were acquired using a 160-channel (axial gradiometers) whole-head KIT MEG system (Model PQ1160R-N2, KIT, Kanazawa, Japan) at the KIT-Macquarie Brain Research Laboratory (Sydney, Australia). Recordings were collected with a 1000 Hz sampling rate and filtered online between 0.03 and 200 Hz. Participants lay in a supine position inside the MEG scanner within a magnetically shielded room (Fujihara Co. Ltd, Tokyo, Japan). Head position was tracked using five marker coils placed on a fitted elastic cap worn on the participant’s head. A photodiode tracked the exact onset of each visual stimulus and was activated by the presentation of a small white square on the screen during each stimulus presentation.

### MEG experimental design

The experimental script was written in MATLAB using functions from the Psychtoolbox^44–46^. Visual stimuli were presented in the MEG via a projector at 114 cm viewing distance and subtended 4° of visual angle. The 96 visual stimuli were presented in random order in 6 runs for each subject. Each run contained 4 repeats of each of the 96 stimuli, for a total of 384 trials per run and 2304 trials in total (24 repeats of each stimulus). A break occurred halfway through each run and participants pressed a key when they were ready to proceed. Images were presented in the center of the screen for 200 ms on a mid-gray background. As for the fMRI experiment, eye movements were not recorded, however, the brief stimulus duration prevented any substantial eye movements during stimulus presentation. The interstimulus interval was jittered and randomly varied between 1–1.5 s on each trial. Each image was presented tilted slightly by 3° to the left or right of center (Fig. 1c) and participants’ task was to report the direction of tilt with a keypress on an MEG-compatible button response pad. The tilt of each stimulus was carefully counterbalanced within and across runs so that tilt direction and/or motor response would not be an informative cue for multivariate pattern analysis. Averaged across participants and runs, the mean task accuracy was 93.2% (SD = 4.8%).

### MEG preprocessing

MEG analysis was conducted using MATLAB (The MathWorks), including functions from the CoSMoMVPA^26^ and FieldTrip^56^ toolboxes. We performed minimal preprocessing on the data^17,57^. Trials were downsampled to 200 Hz (5 ms) temporal resolution and for each trial an epoch from −100 to 1000 ms relative to the onset of the visual stimulus was used for analysis. Principal components analysis was performed to reduce the dimensionality of the data, and the components explaining 99% of the variance were retained for each participant’s dataset. No further preprocessing was conducted on the data prior to analysis^17^.

### MEG multivariate pattern analysis

All analyses were conducted on the principal components across all 160 sensors individually for each subject in sensor-space, with the results then pooled. For cross-decoding we used a leave-one-exemplar-out cross-validation approach. An LDA classifier was trained to discriminate between two of the three categories (e.g., human faces vs. objects) on all but one exemplar of each category (i.e., *n* = 31 images per category). We chose LDA for MEG decoding based on the finding that SVM and LDA produce comparable results for MEG data using the PCA preprocessing pipeline we apply here, yet LDA is generally faster and thus suited to handling MEG timeseries data^17^. This process was repeated in an iterative manner across each cross-validation fold so that each combination of exemplars was used as test data. Classifier accuracy was averaged over each cross-validation fold, and then averaged across participants (Fig. 5a). Statistical significance was assessed using Threshold-Free Cluster Enhancement as implemented in the CoSMoMVPA^26^ toolbox.

RDMs for each time point (from −100 ms before stimulus onset to 1000 ms after stimulus onset, in 5 ms increments) and participant were constructed by correlating the whole-brain patterns across sensors (after PCA) for each pair of stimuli, and taking 1-correlation (Spearman’s rho) to convert to a dissimilarity measure^18,19^. The mean of individual participants’ RDMs produced one RDM per time point (Fig. 5b**;** Supplementary Movie 1). MDS was performed using the MATLAB function *cmdscale* on the average RDM across participants for each time point, with the first two dimensions plotted for visualization (Fig. 5c; Supplementary Movie 2). Comparison between the time-varying MEG RDM and behavioral, visual feature, and saliency RDMs (Fig. 6f) was made by correlating the RDMs using Kendall’s tau-a^25^. This approach was also used for the fMRI-MEG fusion^27^ in Fig. 7.

### Reporting summary

Further information on research design is available in the Nature Research Reporting Summary linked to this article.

## Supplementary information

Supplementary Information

Peer Review File

Supplementary Movie 1

Supplementary Movie 2

Reporting Summary

## Data Availability

The original experimental stimuli and the empirical RDMs (fMRI, MEG, behavioral, output of computational models) are publicly available at the Open Science Framework website for this project: https://osf.io/9g4rz. Source data are provided with this paper.

## Source data

Source Data

## Code availability

The code for these experiments is available from the corresponding author upon request.
