# Supplementary material for: Rapid and dynamic processing of face pareidolia in the human brain
Source: Nat Commun. 2020 Sep 9;11:4518. doi: 10.1038/s41467-020-18325-8 (PMC7481186; doi:10.1038/s41467-020-18325-8)
Supplement: Supplementary file 1 — Supplementary Information [file 41467_2020_18325_MOESM1_ESM.docx]

SUPPLEMENTARY INFORMATION

**Rapid and dynamic processing of face pareidolia in the human brain**

**Wardle et al.**


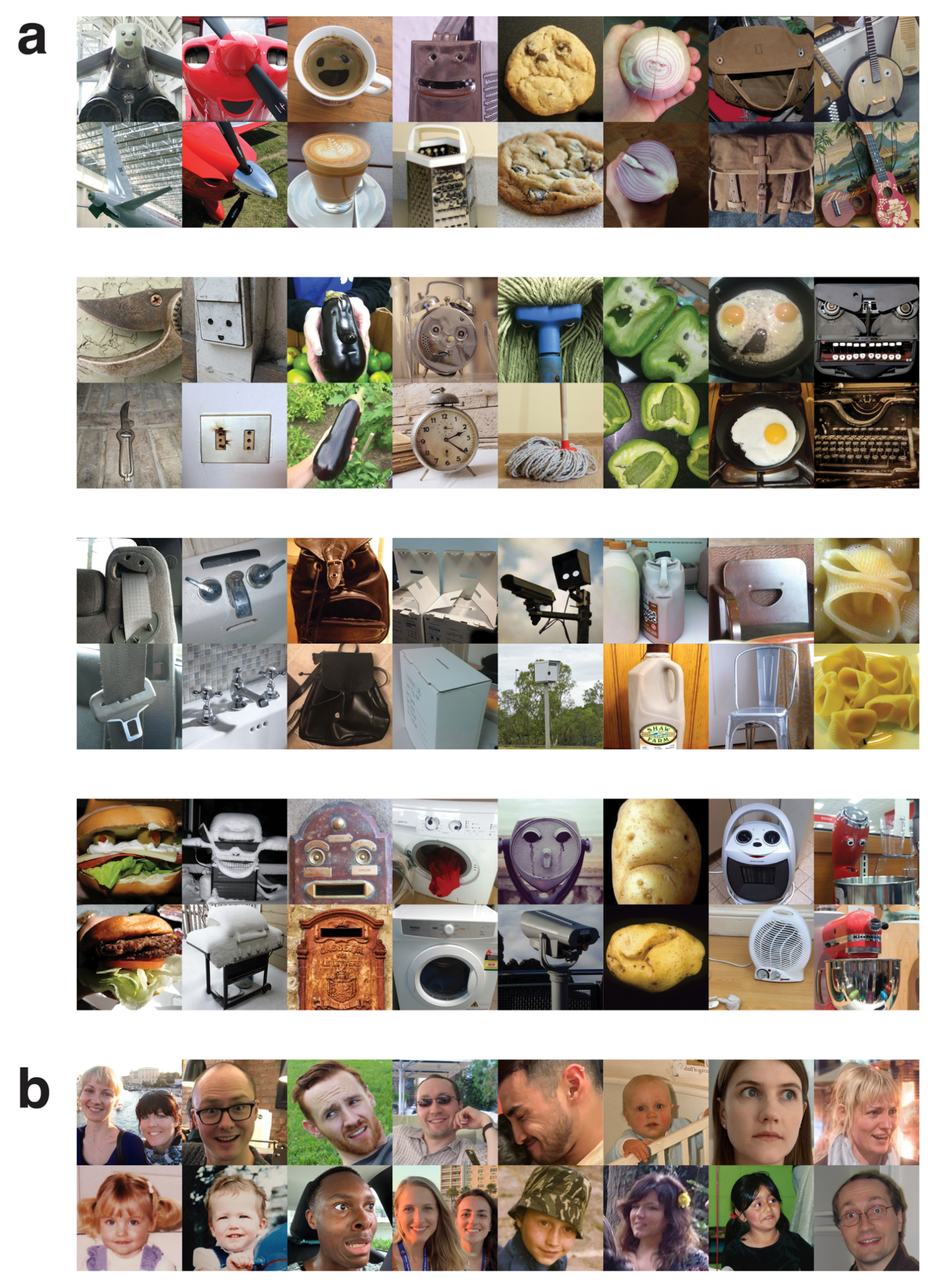


**Supplementary Figure 1.** The stimuli used in the fMRI, MEG, and online behavioral experiments. (a) For each of the 32 objects with illusory faces, we sourced a similar object without a face. (b) 32 human faces provided a comparison for the illusory faces. Note that the human face images used in the experiments are not shown in the figure because we do not have the rights to publish them. The original face stimuli used in the experiments are available at the Open Science Framework website for this project: https://osf.io/9g4rz [http://dx.doi.org/10.17605/OSF.IO/9G4RZ]. The 16 example human faces shown in this figure are similar photographs taken of lab members who gave permission to publish their identifiable images. All stimuli are available at the Open Science Framework website for this project: https://osf.io/9g4rz [http://dx.doi.org/10.17605/OSF.IO/9G4RZ].


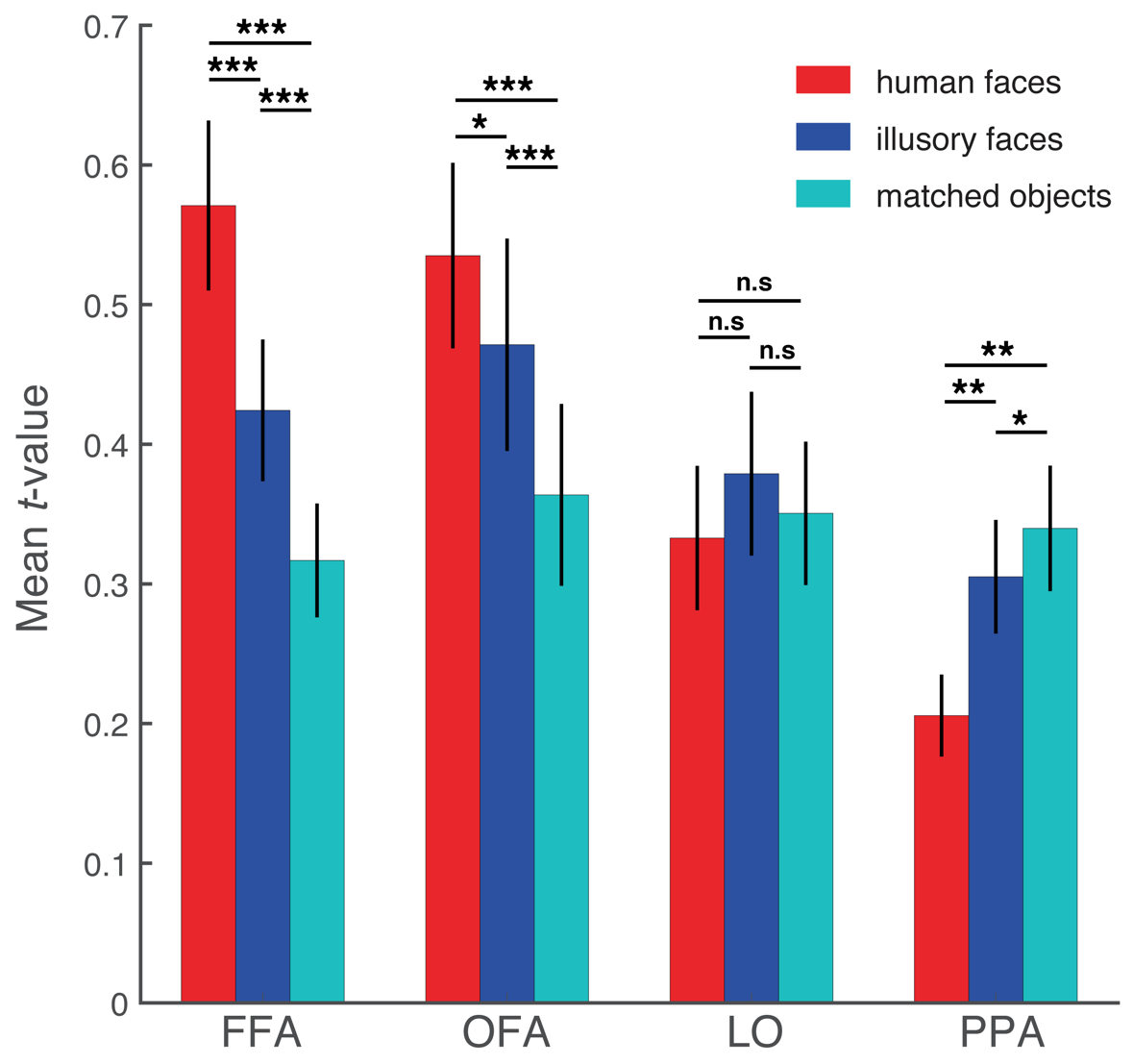


**Supplementary Figure 2**. fMRI univariate results in category-selective ROIs. The mean t-value in each ROI (averaged across voxels) for each stimulus category is shown, averaged across exemplars and N=16 participants. Error bars are SEM. Paired sample t-tests (two-tailed) were conducted using the FDR correction (Benjamini and Hochberg, 1995) to control for multiple comparisons across all ROIs and category pairs. Asterisks indicate statistically significant differences, evaluated using FDR adjusted p-values < .05 (*), < .01 (**), and < .001 (***). Human faces elicited a stronger response than matched objects in all ROIs (FFA: t_(15)_ = 7.65, p = 0.000009, d = 1.91, 95% CI: 0.18, 0.32; OFA: t_(15)_ =10.23, p = 0.0000004, d = 2.56, 95% CI: 0.14, 0.21; PPA: t_(15)_ =-4.19, p = 0.002, d = -1.05, 95% CI: -0.20, -0.07) except for LO (t_(15)_ =-0.80, p = 0.43, d = -0.20, 95% CI: -0.06, 0.03). Illusory faces had a stronger response than objects in face-selective FFA (t_(15)_ = 5.19, p = 0.0003, d = 1.30, 95% CI: 0.06, 0.15) and OFA (t_(15)_ = 6.91, p =0.00002, d = 1.73, 95% CI: 0.07, 0.14), and a weaker response than objects in PPA (t_(15)_ = -2.68, p = 0.02, d = -0.67, 95% CI: -0.06, -0.0071), with no significant difference in LO (t_(15)_ = 2.03, p = 0.07, d = 0.51, 95% CI: -0.0015, 0.06). Conversely, illusory faces produced a weaker response in face-selective FFA (t_(15)_ = -4.75, p = 0.0006, d = -1.19, 95% CI: -0.21, -0.08) and OFA (t_(15)_ = -3.08, p = 0.01, d = -0.7702, 95% CI: -0.11, -0.02) compared to human faces, and a stronger response than real faces in scene-selective PPA (t_(15)_ =3.59, p = 0.005, d = 0.90, 95% CI: 0.04, 0.16), with no significant difference in LO (t_(15)_ =1.72, p = 0.12, d = 0.4303, 95% CI: -0.01, 0.10). Source data are provided as a Source Data file.


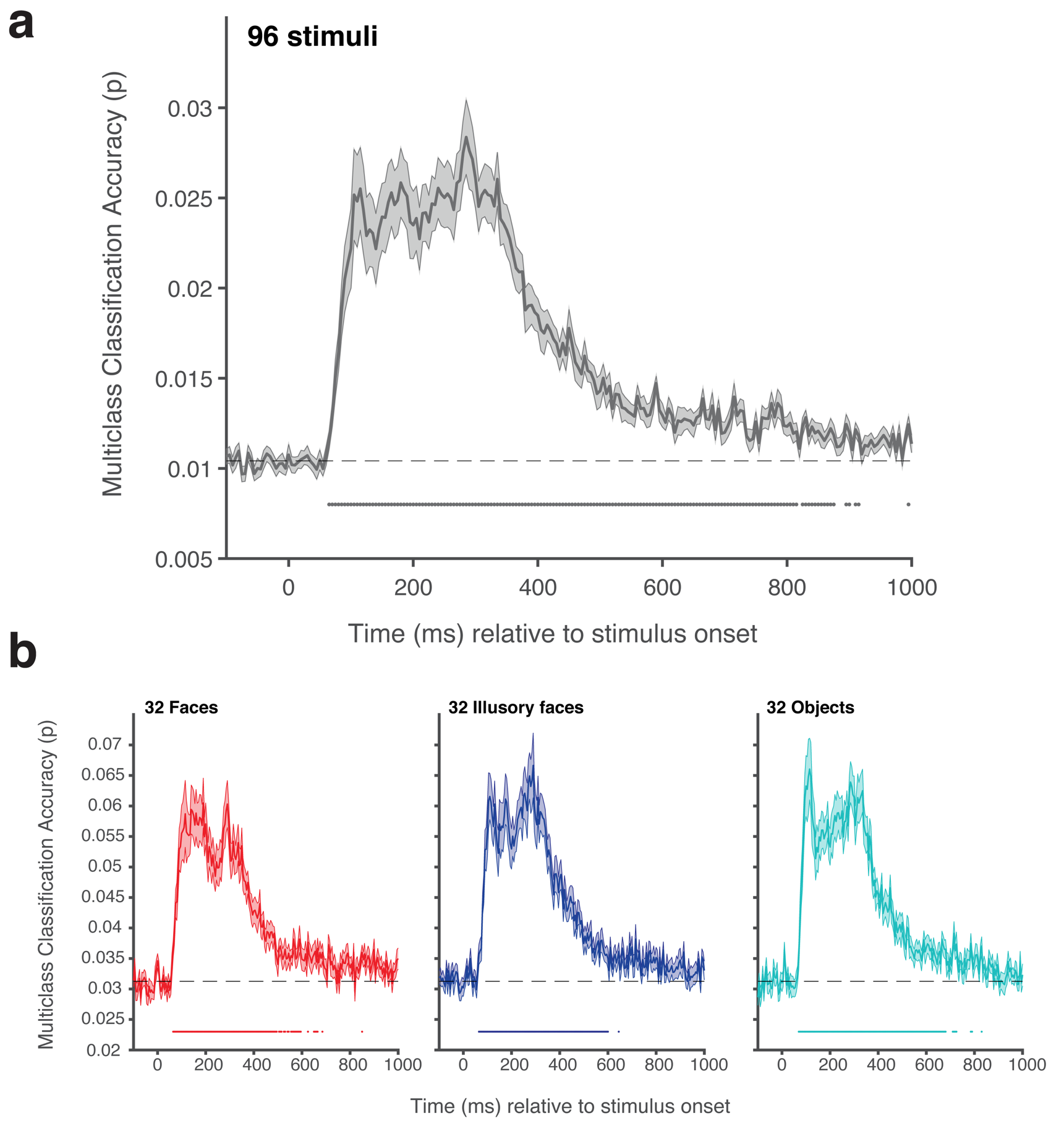


**Supplementary Figure 3.** MEG decoding results at the exemplar level using multiclass classification. (a) Mean classification accuracy for decoding all 96 stimuli using six-fold cross-validation, averaged over N=22 participants. Chance proportion correct (1/96 = 0.01) is marked by the dashed line. Shaded area represents SEM. Multiple comparisons were controlled for using Threshold-Free Cluster Enhancement as implemented in CoSMoMVPA^20^. (b) Mean classification accuracy for decoding stimuli from the three categories (faces, illusory faces, and objects) separately using six-fold cross-validation, averaged over N=22 participants. Chance proportion correct for each category (1/32 = 0.03) is marked by the dashed line. Shaded area represents SEM. Multiple comparisons were controlled for using Threshold-Free Cluster Enhancement. Source data are provided as a Source Data file.
