# Supplementary material for: Rapid and dynamic processing of face pareidolia in the human brain
Source: Nat Commun. 2020 Sep 9;11:4518. doi: 10.1038/s41467-020-18325-8 (PMC7481186; doi:10.1038/s41467-020-18325-8)
Supplement: Supplementary file 5 — Reporting Summary [file 41467_2020_18325_MOESM5_ESM.pdf]

nature research

Corresponding author(s): Susan G Wardle

Last updated by author(s): Aug 3, 2020

# Reporting Summary

Nature Research wishes to improve the reproducibility of the work that we publish. This form provides structure for consistency and transparency in reporting. For further information on Nature Research policies, see Authors & Referees and the Editorial Policy Checklist.

## Statistics

For all statistical analyses, confirm that the following items are present in the figure legend, table legend, main text, or Methods section.

| n/a | Confirmed | |
|---|---|---|
| ☐ | ☒ | The exact sample size ($n$) for each experimental group/condition, given as a discrete number and unit of measurement |
| ☐ | ☒ | A statement on whether measurements were taken from distinct samples or whether the same sample was measured repeatedly |
| ☐ | ☒ | The statistical test(s) used AND whether they are one- or two-sided<br>*Only common tests should be described solely by name; describe more complex techniques in the Methods section.* |
| ☒ | ☐ | A description of all covariates tested |
| ☐ | ☒ | A description of any assumptions or corrections, such as tests of normality and adjustment for multiple comparisons |
| ☐ | ☒ | A full description of the statistical parameters including central tendency (e.g. means) or other basic estimates (e.g. regression coefficient) AND variation (e.g. standard deviation) or associated estimates of uncertainty (e.g. confidence intervals) |
| ☐ | ☒ | For null hypothesis testing, the test statistic (e.g. $F$, $t$, $r$) with confidence intervals, effect sizes, degrees of freedom and $P$ value noted<br>*Give P values as exact values whenever suitable.* |
| ☒ | ☐ | For Bayesian analysis, information on the choice of priors and Markov chain Monte Carlo settings |
| ☒ | ☐ | For hierarchical and complex designs, identification of the appropriate level for tests and full reporting of outcomes |
| ☐ | ☒ | Estimates of effect sizes (e.g. Cohen's $d$, Pearson's $r$), indicating how they were calculated |

*Our web collection on statistics for biologists contains articles on many of the points above.*

## Software and code

Policy information about availability of computer code

| | |
|---|---|
| Data collection | The experimental code for stimulus presentation was written in MATLAB (v2019a, The MathWorks) using functions from the Psychtoolbox (v3). |
| Data analysis | The fMRI data was analyzed using AFNI (v19.0.25) and SUMA, MATLAB (v2019a, The MathWorks), Freesurfer (v6.0), The Decoding Toolbox (v3.996) and custom code. The MEG data was analyzed using MATLAB (v2019a, The MathWorks), including functions from the CoSMoMVPA and FieldTrip toolboxes, and custom code. |

For manuscripts utilizing custom algorithms or software that are central to the research but not yet described in published literature, software must be made available to editors/reviewers. We strongly encourage code deposition in a community repository (e.g. GitHub). See the Nature Research guidelines for submitting code & software for further information.

## Data

Policy information about availability of data

All manuscripts must include a data availability statement. This statement should provide the following information, where applicable:

- Accession codes, unique identifiers, or web links for publicly available datasets
- A list of figures that have associated raw data
- A description of any restrictions on data availability

The original experimental stimuli and the empirical RDMs (fMRI, MEG, behavioral, output of computational models) are publicly available at the Open Science Framework website for this project: https://osf.io/9g4rz (DOI: 10.17605/OSF.IO/9G4RZ) Source data are provided with this paper.

# Field-specific reporting

Please select the one below that is the best fit for your research. If you are not sure, read the appropriate sections before making your selection.

☐ Life sciences ☒ Behavioural & social sciences ☐ Ecological, evolutionary & environmental sciences

For a reference copy of the document with all sections, see nature.com/documents/nr-reporting-summary-flat.pdf

# Behavioural & social sciences study design

All studies must disclose on these points even when the disclosure is negative.

| | |
|---|---|
| Study description | This study measured the response of the human brain to illusory faces in objects using fMRI and MEG. |
| Research sample | Human participants for the neuroimaging experiments were volunteers from the university community of Macquarie University in Sydney, Australia. Human participants in the online behavior experiment were volunteers sampled from the general US population using Amazon MechanicalTurk. 22 participants (8 male, 14 female, mean age 26.2 years, range 18-41 years) completed the MEG experiment. 21 participants (11 male, 10 female, mean age 25.4 years, range 20-36 years) participated in the fMRI experiment. N=4 participants were removed from the fMRI analysis due to inability to define category-selective regions from their localizer data and N=1 was excluded due to failing to complete the experiment, leaving N=16 fMRI datasets for analysis. 20 participants (12 female, 7 male, 1 other) completed the behavioral experiment online via Amazon MechanicalTurk. |
| Sampling strategy | Human volunteers were recruited for the experiments and provided informed consent. We recruited a similar number of participants for each experiment (N=22 MEG, N=21 fMRI, N=20 behavior) based on standard sample sizes in the field. |
| Data collection | The neuroimaging experiments used MRI and MEG scanners and were conducted by authors SGW and LT. The behavioral experiment was conducted online using Amazon Mechanical Turk by SGW. The study used a within-subjects design, thus all participants completed all experimental conditions. |
| Timing | fMRI experiment: start 11-Oct-2017, end 01-Nov-2017. MEG experiment: start 19-Oct-2017, end 12-Dec-2017. Amazon Mechanical Turk behavior experiment: start 21-Aug-2018, end 21-Aug-2018. |
| Data exclusions | No data were excluded from the MEG or behavioral experiments. Out of 21 original participants in the fMRI experiment, N=4 participants were removed from the fMRI analysis post-hoc due to inability to define category-selective regions from their localizer data and N=1 was excluded due to failing to complete the experiment, leaving N=16 fMRI datasets for analysis. |
| Non-participation | N=1 discontinued the fMRI experiment due to discomfort. |
| Randomization | Participants were not allocated to groups, a within-subjects design was used for all experiments. |

# Reporting for specific materials, systems and methods

We require information from authors about some types of materials, experimental systems and methods used in many studies. Here, indicate whether each material, system or method listed is relevant to your study. If you are not sure if a list item applies to your research, read the appropriate section before selecting a response.

## Materials & experimental systems

| n/a | Involved in the study | |
|---|---|---|
| ☒ | ☐ | Antibodies |
| ☒ | ☐ | Eukaryotic cell lines |
| ☒ | ☐ | Palaeontology |
| ☒ | ☐ | Animals and other organisms |
| ☐ | ☒ | Human research participants |
| ☒ | ☐ | Clinical data |

## Methods

| n/a | Involved in the study | |
|---|---|---|
| ☒ | ☐ | ChIP-seq |
| ☒ | ☐ | Flow cytometry |
| ☐ | ☒ | MRI-based neuroimaging |

## Human research participants

Policy information about studies involving human research participants

| | |
|---|---|
| Population characteristics | All participants were healthy human volunteers. 22 participants (8 male, 14 female, mean age 26.2 years, range 18-41 years) completed the MEG experiment. 21 participants (11 male, 10 female, mean age 25.4 years, range 20-36 years) participated in the fMRI experiment. 20 participants (12 female, 7 male, 1 other, age not collected) completed the behavioral experiment online via Amazon MechanicalTurk. |

| | |
|---|---|
| Recruitment | Participants for neuroimaging experiments (fMRI, MEG) were recruited on-campus at Macquarie University. Participants in the behavioral experiment were recruited online via Amazon MechanicalTurk. |
| Ethics oversight | All imaging experiments were approved by the Human Research Ethics Committee of Macquarie University. The online experiments were conducted on Amazon MechanicalTurk following guidelines set by the NIH Office of Human Subjects Research Protections (OHSRP). |

Note that full information on the approval of the study protocol must also be provided in the manuscript.

# Magnetic resonance imaging

## Experimental design

| | |
|---|---|
| Design type | task event-related design (for experimental runs) and block design (for localizer runs) |
| Design specifications | The fMRI experiment consisted of a structural anatomical scan, 2 functional localizer runs, and 7 experimental runs. Localizer runs began with a 4s fixation period before the first stimulus block. For each stimulus class, there were 3 unique blocks of 18 images. Each of the 20 images within a block (18 unique + 2 repeats) was shown for 600ms followed by a 200ms inter- stimulus interval. The 16s stimulus blocks alternated with 10s fixation blocks. Each of the 4 stimulus categories was repeated three times per 5-minute localizer run, once per unique image set. In the experimental runs, each of the 96 stimuli was shown once per run, in random order. In each 4 sec trial, stimuli were presented for 300 ms at the start of the trial on a gray background. For the remaining time in each trial after stimulus offset, a gray background with a fixation cross was shown. In addition to the 96 stimulus trials, 32 blank null trials (4 sec duration each) were inserted randomly in the sequence for each run and each participant. Finally, 4 null trials were inserted at the start and end of each run. This produced 136 trials total per run for a run duration of 9 mins. |
| Behavioral performance measures | A task unrelated to face detection was used in order to maintain participants' alertness during the fMRI experimental runs. Each image was presented tilted slightly by 3° to the left or right of center and participants' task was to report the direction of tilt with a keypress. fMRI mean task accuracy was 92.5% (SD = 8.6%). For the localizer runs, participants performed a 1-back task, pressing a key each time an image was repeated twice in a row. Every time a block was run, the images were presented in a random order and two random images were repeated twice for the 1-back task. |

## Acquisition

| | |
|---|---|
| Imaging type(s) | MPRAGE and EPI |
| Field strength | 3T |
| Sequence & imaging parameters | A high-resolution T1-weighted structural MRI scan (3D-MPRAGE sequence, 1 x 1 x 1 mm voxel size, in-plane matrix size: 256 x 256, 176 slices, TR=2 s, TE=2.36 ms, FA=9 deg) was collected for each participant at the start of the session. Functional scans were acquired with a 2D T2*-weighted EPI acquisition sequence: TR = 2.5 s, TE = 32 ms, FA = 80 deg, voxel size: 2.8 x 2.8 x 2.8 mm, in plane matrix size: 92 x 92. A whole-brain volume containing 42 slices was collected. 128 volumes were collected per localizer run (5 mins each), and 218 per experimental run (9 mins each). |
| Area of acquisition | Whole brain |

Diffusion MRI ☐ Used ☒ Not used

## Preprocessing

| | |
|---|---|
| Preprocessing software | fMRI data was preprocessed using the AFNI software package. |
| Normalization | All analyses were conducted in the native brain space of each participant. |
| Normalization template | The data were not normalized. |
| Noise and artifact removal | EPIs were slice-time corrected, motion-corrected, and co-registered to the participant's individual anatomical volume. Spatial smoothing of 4mm full-width at half- maximum was applied to the localizer runs only, no smoothing was conducted on the experimental runs. |
| Volume censoring | None applied |

## Statistical modeling & inference

| | |
|---|---|
| Model type and settings | Data from the two independent localizer runs were entered as factors into a GLM in AFNI to estimate the beta weights for faces, scenes, objects, and scrambled objects. Contrasts of faces–scenes, scenes–faces and objects–scrambled objects produced t-maps used to define the boundaries of each ROI. For experimental runs, beta weights were estimated in a GLM using AFNI for each of the 96 stimuli, producing a separate beta weight for each run (i.e. 7 beta weights per stimulus) |
| Effect(s) tested | Decoding and representational similarity analysis were used to determine brain regions sensitive to human faces, illusory faces, and matched objects. |

Specify type of analysis: ☐ Whole brain ☐ ROI-based ☒ Both

| Anatomical location(s) | The ROI based analysis included functionally-defined face-selective regions FFA and OFA, scene selective PPA, and object-selective LO. A whole-brain decoding searchlight was also conducted. |
|---|---|
| Statistic type for inference (See Eklund et al. 2016) | Statistical significance for the fMRI decoding analysis was assessed using one-tailed t-tests on the decoding accuracy for each ROI. |
| Correction | Multiple comparisons were controlled for the fMRI decoding analysis (at alpha < .05) using FDR. |

## Models & analysis

| n/a | Involved in the study |
|---|---|
| ☒ | ☐ Functional and/or effective connectivity |
| ☒ | ☐ Graph analysis |
| ☒ | ☐ Multivariate modeling or predictive analysis |
