# Supplementary material for: Rapid and dynamic processing of face pareidolia in the human brain
Source: Nat Commun. 2020 Sep 9;11:4518. doi: 10.1038/s41467-020-18325-8 (PMC7481186; doi:10.1038/s41467-020-18325-8)
Supplement: Supplementary file 2 — Peer Review File [file 41467_2020_18325_MOESM2_ESM.docx]

Peer Review Information

**Journal:** Nature Communications

**Manuscript title: Rapid and dynamic processing of face pareidolia in the human brain**

**Corresponding author name(s):** Dr Susan Wardle

**Editorial notes:**

**Transferred manuscripts**

This manuscript has been previously reviewed at another journal that is not operating a transparent peer review scheme. This document only contains reviewer comments and rebuttal letters for versions considered at *Nature Communications*.

**Reviewer comments & decisions:**

**Reviewer comments, first version:**

Reviewer #1 (Remarks to the Author):

In the manuscript entitled ‘Rapid and dynamic processing of face pareidolia in the human brain’, the authors Wardle, Taubert, Teichmann & Baker investigate whether face pareidolia originates from rapid visual feature analysis or slower cognitive interpretations of face-like objects. The main result of the study is that there is a transformation at around 250 ms regarding how illusory face information is processed – at first illusory faces are processed similarly as real faces, but later they are processed similarity as visual objects. The manuscript is clearly written, the topic is interesting and relevant and the results provide novel insights into face processing. However, I did notice several issues that needs clarification. Please my detailed comments below.

1. The main result of the paper, as stated in the abstract was: "Illusory faces were initially represented similarly to real faces, but within ~250ms, the representation transformed, and they became more similar to ordinary objects." The latter results (similar processing of illusory faces and objects at 250 ms) was very clear and evident, e.g., in Figure 4. The former results was not as clear (illusory faces and real faces represented similarly). In Figure 4a, the early classification accuracy between faces vs. illusory faces was higher than the classification accuracy between illusory faces vs. objects. Thus, illusory faces clearly differed from objects, but saying that they were processed similarly to real faces is not correct, or at least, it’s an exaggeration or over-simplification of the results. Similar processing of faces and illusory faces would also suggest that high similarity between faces and illusory faces, and high dissimilarity between all faces and objects. Looking at the RMDs in Figure 4b (or supplementary videos) shows that this was not the case, however. Could the authors elaborate/clarify the early similarity of processing faces and illusory faces?

2. One of the main goals of the manuscript is to combined MEG and fMRI data, and to differentiate ‘perceptual’ and ‘cognitive’ explanations of face pareidolia. The former explanation predicts early processing at the face specific regions, and the latter late processing at the category selective cortex. I was a bit disappointed for not finding searchlight results combining MEG and fMRI data. Currently, the MEG and fMRI data is combined only for 2/3 of stimuli in four ROIs. If the authors’ conclusion is correct, i.e., that there is a change in processing of illusory faces, then different spatio-temporal clusters would be expected for real faces, illusory faces, and visual objects. The data could be split into three (faces, illusory faces, and objects) and then searchlight MEG-fMRI could be conducted, and these should show different spatio-temporal flow of information from primary visual cortex to face specific and/or category selective cortex. Previous studies using combined EEG/MEG-fMRI have shown the flow of information in visual cortex separately for visual objects (Cichy, Pantazis et al., Nature Neuroscience, 2014) and for faces (Muukkonen, Ölander et al, bioRxiv, 2019). The data set in the current manuscript provides an interesting opportunity to compare the information flow of different stimulus categories.

3. The dissimilarity/distance metrics that the authors used was not consistent in all analyses. In the decoding analyses, the metric was classification accuracy and in the RSA it was 1-correlation. The cross-validated Mahalanobis distance is often preferred (Walther, Nili et al., NeuroImage, 2016) over the correlation based distance metrics. The RDMs for RSA could also be constructed with pair-wise decodings. Have the authors tested different distance metrics, does the results hold if 1-correlation is changed to cross-validated Mahalanobis distance (or classification accuracy)?

4. One of the aims of the study was to find whether face pareidolia is perceptual effect or more cognitive effect, requiring reinterpretation of the visual image. However, the stimulus duration was 200/300 ms, and the inter-stimulus interval in MEG quite short. The subjects did not have much time to reinterpret the images, so does the experimental design encourage
the subjects to use rapid processing of visual features?

5. The fMRI decoding was done with leave-one-exemplar-out cross-validation. Leave-one-run-out cross-validation is sometimes preferred in fMRI to ensure full independence of the training and test sets. Since the beta coefficients for all stimuli are based on same GLM, there might be some dependencies between the stimuli in the same runs. Did the authors take this into account? Or was similar results obtained with leave-one-run-out cross-validation?

6. Only two models are applied, the saliency model than the behavioral rating model. Since the higher-level processes related to face and object recognition, are likely invariant to retinotopic location, it is not surprising that the saliency model does not explain much of the measured responses. A better prediction could be computed by first extracting the objects/faces from the stimulus images, aligning them spatially (so that eyes and mouths overlap), and then computing the model predictions. Or a Gabor filter or wavelet pyramid model could also be applied to the (spatially aligned) images. It is known that Gabor filtering model best explains the fMRI responses to faces (Carlin, Kriegeskorte, Plos Computational Biolory, 2017).

7. The main advantage of MVPA analyses is that brain responses for each individual stimulus can be compared to each other. In the decoding analyses, however, the authors collapse the data to three categories. Was it necessary to combine all samples of each category, or was the decoding also successful in individual pairs of samples or different categories?

8. Univariate vs. multivariate responses. It would be interesting to see what the multivariate analyses add to univariate responses. Was the mean signal changes in the four ROIs similar for all three categories, or did univariate results also show differences between categories. Could the authors add a notion of univariate results to the manuscript?

9. SVM classification was used for fMRI, but LDA for MEG. What was the reason for using different method? For methodological consistency, the same method for MEG and fMRI should be preferred.

10. Almost 25% percent of measured fMRI data was rejected (5/21 subjects). Four subjects were rejected due to the amount of movement. What was the criterion for rejection (how much movement), and was the criterion decided before the measurements?

11. The author emphasize the importance of yoked design, and controlling for visual features between objects and illusory faces. The real faces were not, however, yoked to the illusory faces. Is there any specific reason for this? Of course it is more difficult, but for example, the red front view of a plane could have been paired with an image of face and red clothes; green kiwi fruit with face and green clothes etc. The authors say that “Human faces were not individually matched to the illusory faces because of the ambiguity in defining parameters such as age, race and gender for illusory faces.” But colors, sizes, spatial positions, orientations can be matched.

12. While reading the manuscript, it was not always clear if statistical tests were corrected for multiple comparison and whether the same method for correction was always used. Please add, at least to figure captions, more details on statistics and corrections for multiple comparisons.

13. “In order to maintain attention… the image on each trial was rotated slightly to the left or right by 3°….” Does this maintain attention to stimuli, or direct attention to sides of the stimuli? Was fixation of gaze restricted center of the screen or monitored? What was the performance (mean correct) in the orientation task?

14. The face stimuli “was selected to have a high degree of variance across age, gender, race, expression… in order to match the high variance of the illusory face..” Was this matched variance quantified somehow?

15. Behavioral rating was rating for ‘face-ness’, and ratings differ between objects and illusory faces. Did the authors consider the other way around, rating for ‘object-ness’, would illusory faces differ from objects with object-ness rating task?

16. “However, we did match the number of images containing more than one face across human and illusory face sets (N = 2).” I don’t understand what the N=2 refers to, a typo? Two illusory face sets? Two images that contain more than one face? Two participants matching the stimuli?


Reviewer #2 (Remarks to the Author):

The study examines the neural profile of face detection through the joint use of fMRI and MEG as applied to illusory face perception. Specifically, it investigates the cortical locus and the temporal dynamics of pareidolia as a means to characterize the representational basis of face detection. Based on the rapid but short-lived assimilation of illusory faces to faces and to the confinement of this result to occipitotemporal face-selective regions the authors argue for a broadly-tuned template for face detection. The use of pareidolia as a window into the neural basis of face detection is certainly appealing and the results are in broad agreement with current understanding of face processing. The logic of the study is relatively straightforward and the results provide an informative contribution to the topic. My questions below mostly target aspects of the study that could benefit from further clarification.

1. The authors claim that “the representation of illusory faces was confined to occipital-temporal face-selective visual cortex” (abstract) but there is no investigation of more anterior face-selective regions (e.g., anterior temporal or inferior frontal areas). A more thorough investigation of such areas or a whole-brain analysis using searchlight could be informative here.
2. It is stated that “by 160ms, the illusory faces are more distinct from the matched objects and closer to the human faces compared to the earlier and later timepoints” (p. 9). However, an examination of Fig. 4c shows illusory faces to be less intermingled with faces at 160s (t2) compared to 130ms (t1). Please clarify.
3. The current use of saliency is rather problematic as a means to investigate a presumed face template. Two illusory face images could easily generate similar maps that do not represent such a template. Also, the correlation with MEG-based similarity could arise simply from low-level image similarity across illusory face images (and it would also be expected to peak early as saliency does in Fig. 5b). Perhaps a more direct test would be to correlate illusory face maps directly to face saliency maps and treat the resulting estimate(s) by analogy with face ratings in the same section/figure. This would eliminate the issue above and would also render the two analyses in Fig. 5b more immediately comparable.
4. The correlation with saliency (Supplementary Fig. 2) exhibits rather different time courses for different models: both DeepGaze2 and GIST show significance even after 400ms, unlike Itti-Koch, and GIST shows no clear peak. If the authors retain this analysis (please see point 3 above) please discuss this divergence.
5. As the authors note, “the percept of a face persists well beyond the initial few hundred milliseconds” (p. 16). Yet, the discrimination of illusory faces from faces returns to chance before 1s, unlike the other two types of discrimination (Fig. 4a). What supports the perception of such images as ‘face-like’ at a later time?
6. The authors use different classifiers for fMRI and MEG data, linear SVM and LDA. Presumably this is not because SVM was not successful with MEG data. Please explain.

A few smaller issues:
- when displaying representational dissimilarity matrices (e.g., in Fig 4b) it could be useful to specify the limits of the scales rather than just ‘min’ and ‘max’
- what threshold was used for functional ROI definition and how large were the resulting ROIs (in number of voxels)?
- the current results are in relative agreement with previous studies that applied reverse correlation to derive broadly-tuned templates for face detection from MEG, EEG and fMRI signals (Smith, Fries, Gosselin et al, 2009, Cereb Cortex; Smith, Gosselin & Schyns, 2012, Curr Biol; Nestor, Vettel & Tarr, 2013, Hum Brain Mapp) and this convergence would be relevant to note
- please specify what error bars (in Fig 2b) and shaded areas (e.g., in Fig 4a) represent (standard errors, confidence intervals etc.)

**Author rebuttal, first version:**

Reviewer #1 (Remarks to the Author):

In the manuscript entitled ‘Rapid and dynamic processing of face pareidolia in the human brain’, the authors Wardle, Taubert, Teichmann & Baker investigate whether face pareidolia originates from rapid visual feature analysis or slower cognitive interpretations of face-like objects. The main result of the study is that there is a transformation at around 250 ms regarding how illusory face information is processed – at first illusory faces are processed similarly as real faces, but later they are processed similarity as visual objects. The manuscript is clearly written, the topic is interesting and relevant and the results provide novel insights into face processing. However, I did notice several issues that needs clarification. Please my detailed comments below.

1. The main result of the paper, as stated in the abstract was: "Illusory faces were initially represented similarly to real faces, but within ~250ms, the representation transformed, and they became more similar to ordinary objects." The latter results (similar processing of illusory faces and objects at 250 ms) was very clear and evident, e.g., in Figure 4. The former results was not as clear (illusory faces and real faces represented similarly). In Figure 4a, the early classification accuracy between faces vs. illusory faces was higher than the classification accuracy between illusory faces vs. objects. Thus, illusory faces clearly differed from objects, but saying that they were processed similarly to real faces is not correct, or at least, it’s an exaggeration or over-simplification of the results. Similar processing of faces and illusory faces would also suggest that high similarity between faces and illusory faces, and high dissimilarity between all faces and objects.

Looking at the RMDs in Figure 4b (or supplementary videos) shows that this was not the case, however. Could the authors elaborate/clarify the early similarity of processing faces and illusory faces?

1

We thank the reviewer for highlighting the lack of clarity in our text. What we show is that illusory faces are processed more similarly to real faces *than closely matched non-face objects*.

In the MEG results, this is shown by:

(1) the higher decoding accuracy for human faces vs objects compared to that for human faces versus illusory faces at 160ms (difference between red and gray peaks at *t2* in Fig. 5a) – as illusory faces are more similar to human faces than matched objects at this timepoint, it is harder to decode illusory objects from human faces than matched objects
(2) The RSA/MDS results in Fig 5b & 5c at 260ms, which show illusory faces were more similar to human faces than matched objects were at 160ms.

As pointed out, this is different than claiming that illusory faces are processed the same as real faces – there is still a strong difference between real and illusory faces.

In the conclusion we now acknowledge the difference between real and illusory faces: “In sum, illusory faces are represented uniquely in the brain compared to both real human faces and similar objects that do not have illusory facial features.” (page 19)

We have revised the main text in several places to make this distinction clearer:

-page 2 (Abstract)

-page 3 (Introduction)

-new paragraph on page 10-11 (Results)

-page 18 (Discussion)

-new paragraph on page 19 (Discussion).

New text on page 10-11:

“The most striking feature of the representational structure is how it changes dynamically across these three

timepoints, as visualized in the dissimilarity matrices (**Fig. 5b**) and MDS plots **(Fig. 5c**). At 130 ms the human faces are already more similar to each other than to illusory faces or objects (**Fig. 5b**), as shown by the clustering of the human face exemplars in the MDS plot (**Fig. 5c**). Notably, a few illusory face exemplars are clustered with the human faces (**Fig. 5c**). By 160ms, the illusory faces have started to segregate from the matched objects (**Fig. 5c**), and have become more similar to the human faces than the matched objects are (**Fig. 5b**). There is a categorical shift in the representational organization between 160 and 260 ms: initially the illusory faces are distinct from the matched objects, but only 100 ms later, the illusory faces are grouped with the matched objects (**Fig. 5c**). As with the rapid onset of category-level cross-decoding (**Fig. 5a**), this shows that illusory faces are rapidly processed. These errors of face detection are initially treated more like real faces than matched objects are, however, the human brain rapidly resolves this detection error and they are represented more similarly to objects in less than a quarter of a second.”

New text on page 19:

“Although we observe a rapid “face-like” response to illusory faces in objects, there are substantial differences in the representation of human faces versus illusory faces. The fMRI data showed that in face-selective FFA and OFA, human faces have more similar within-category activation patterns than illusory faces, which are less similar to each other. Further, in non-face areas such as LO and PPA, the illusory faces group with objects rather than human faces. This is similar in the time domain revealed by MEG, in which illusory faces have a more transient “face-like” response than human faces, and are much more similar to nonface objects within a couple hundred milliseconds. The fact that human faces and illusory faces are readily decoded from each other in both fMRI and MEG is also indicative of differences in their representation. Overall, this difference is consistent with the interpretation of illusory faces as a quickly resolved error of a broadly-tuned face detection system^2,3^. Thus although illusory faces elicit a face-like response, they are not represented in the same way as human faces within the visual system.”

2

2. One of the main goals of the manuscript is to combined MEG and fMRI data, and to differentiate ‘perceptual’ and ‘cognitive’ explanations of face pareidolia. The former explanation predicts early processing at the face specific regions, and the latter late processing at the category selective cortex. I was a bit disappointed for not finding searchlight results combining MEG and fMRI data. Currently, the MEG and fMRI data is combined only for 2/3 of stimuli in four ROIs. If the authors’ conclusion is correct, i.e., that there is a change in processing of illusory faces, then different spatio-temporal clusters would be expected for real faces, illusory faces, and visual objects. The data could be split into three (faces, illusory faces, and objects) and then searchlight MEG-fMRI could be conducted, and these should show different spatio-temporal flow of information from primary visual cortex to face specific and/or category selective cortex. Previous studies using combined EEG/MEG-fMRI have shown the flow of information in visual cortex separately for visual objects (Cichy, Pantazis et al., Nature Neuroscience, 2014) and for faces (Muukkonen, Ölander et al, bioRxiv, 2019). The data set in the current manuscript provides an interesting opportunity to compare the information flow of different stimulus categories.

The main goal of the study was to understand the processing of illusory faces. We did that by using both fMRI and MEG as complementary measures of brain activity to take advantage of their superior spatial and temporal resolution respectively. Although fMRI-MEG ‘fusion’ allows these measures to be combined, there are limitations to applying this in a searchlight manner, especially in terms of power (given the issue of multiple comparisons). For our principal fMRI analyses, we used an ROI-based approach to increase power and then applied fMRI-MEG fusion within these ROIs. Note that this is the same approach used by Cichy and colleagues (Nature Neuroscience, 2014). While the approach the reviewer suggests is interesting, dividing our stimuli into three further reduces the amount of power, and does not directly address the critical question of our study, which is the *difference* in processing between illusory faces and non-face objects.

It is this focus on the *difference* in processing between illusory faces and non-face objects that also explains why we excluded real faces from the fMRI-MEG fusion analyses. If we included real faces, they would be the principal driver of the correlation and the results would not tell us anything about the processing of illusory faces. This is shown in the figure below (**Response Figure 1**), which is the same analysis but conducted on all stimuli including human faces. Compared to Fig. 7 in the main text, the results are much stronger here: the RDMs for all four fMRI ROIs significantly correlate with the MEG RDM for most of the time course. However, these plots only tell us that there is a significant correspondence between the fMRI and MEG RDMs. Examination of the fMRI and MEG RDMs suggests this primarily reflects the high within-category similarity for the human faces and lower between-category similarity between faces and either illusory faces or non-face objects, and thus it is not informative about the processing of illusory faces. Rather, we want to know where the *difference* in the processing of illusory faces and non-face objects is reflected both temporally and spatially.

3

|  | **Response Figure 1: fMRI-MEG fusion when human faces (i.e. all 96 stimuli) are included in the analysis** (equivalent of Fig. 7 in the manuscript, in which the human face category is excluded from the analysis).Note that the fMRI-MEG correlation is significant for all four ROIs for most of the time course, suggesting that it is primarily driven by the inclusion of human faces. The version in Fig 7 of the paper shows only a limited correlation with FFA when human faces are removed from the analysis, thus reflecting processing that differentiates between illusory face and non-face objects. |
| --- | --- |

While we think that running a searchlight fMRI-MEG fusion analysis like the reviewer suggested is not fruitful for the current data, we have now conducted an exploratory fMRI decoding searchlight (new Fig. 3 in the revised manuscript) to help validate our choice of ROIs. The results show that maximal decoding for both human faces vs objects and illusory faced versus objects occurs on the ventral surface, overlapping with the predetermined FFA and OFA ROIs. The searchlight provides no evidence for decoding of illusory faces in any other brain regions. We have added this exploratory analysis to the revised manuscript as it is helpful in providing context for our planned ROI analysis.

|  | **new Figure 3: new exploratory searchlight analysis added to revised manuscript.** Panel (a) cross-decoding for human faces versus objects and panel (b) cross-decoding for illusory faces versus objects. |
| --- | --- |

4

3. The dissimilarity/distance metrics that the authors used was not consistent in all analyses. In the decoding analyses, the metric was classification accuracy and in the RSA it was 1-correlation. The cross-validated Mahalanobis distance is often preferred (Walther, Nili et al., NeuroImage, 2016) over the correlation based distance metrics. The RDMs for RSA could also be constructed with pair-wise decodings. Have the authors tested different distance metrics, does the results hold if 1-correlation is changed to cross-validated Mahalanobis distance (or classification accuracy)?

We planned our metrics prior to data collection to be classification accuracy for decoding, and 1-correlation for RSA, which are both standard metrics in the field for both fMRI and MEG multivariate analyses. We did not test any different distance metrics other than what we planned and present in the manuscript, and would not wish to inflate our effective type 1 error rate by doing this. Moreover, the decoding and RSA results are consistent with each other for both the fMRI and MEG data sets, and allow us to draw the same conclusions about the processing of illusory faces.

Note that while Walther and colleagues (2016) used their results to advocate for the cross-validated Mahalanobis distance, the correlation distance performs as well (and in some cases better) especially for the visual datasets with many conditions that are similar to our dataset here. The comparison of different distance metrics is an important topic, but is outside the scope of the aims of this paper.

4. One of the aims of the study was to find whether face pareidolia is perceptual effect or more cognitive effect, requiring reinterpretation of the visual image. However, the stimulus duration was 200/300 ms, and the inter-stimulus interval in MEG quite short. The subjects did not have much time to reinterpret the images, so does the experimental design encourage the subjects to use rapid processing of visual features?

We deliberately used short stimulus presentation times in both fMRI and MEG paradigms to reduce the possibility of eye movements (now added to Method page 22,24). Our data shows a clear difference between the processing of illusory faces and similar non-face objects, indicative that these brief presentation times are sufficient to process the presence of an illusory face. In a separate project (under review) we have shown that illusory faces facilitate visual search in comparison to matched object targets, evidence for rapid processing in a behavioral paradigm.

In terms of the cognitive reinterpretation possibility, we would still expect this to occur on a timescale substantially shorter than one second (which is what we selected as the upper limit on our time window for the MEG analyses for each trial as was built into the experimental design). Our predictions in Fig. 4 use the processing of human faces vs objects as an anchor in time for comparison with the illusory faces, and the corresponding data in Fig 5a show the one second time window is appropriate as maximal decoding has already tapered off within this time window.

5. The fMRI decoding was done with leave-one-exemplar-out cross-validation. Leave-one-run-out cross-validation is sometimes preferred in fMRI to ensure full independence of the training and test sets. Since the beta coefficients for all stimuli are based on same GLM, there might be some dependencies between the stimuli in the same runs. Did the authors take this into account? Or was similar results obtained with leave-one-run-out cross-validation?

We used leave-one-exemplar-out cross validation so that “The classifier's performance was tested on data from stimulus exemplars of each category not used in the training set, thus correct classification required generalization to new stimuli, ruling out an explanation based on visual features associated with specific images in the training set.” (p. 7)

However, the reviewer makes a good point and as suggested we have now rerun the analysis as combined leave-one-exemplar-out and leave-one-run out cross-validation and obtained comparable

5

results to our original analysis. The new analysis did not change the statistical significance of the results or the conclusions. We have replaced the fMRI decoding analysis in the paper with these new results (Figure 2b, statistical results on p. 7).

**New Figure 2b:** fMRI cross-decoding results using combined leave-exemplar-out and leave-one-run-out cross validation. Results are consistent with our original version using leave-exemplar-out only – all comparisons that were statistically significant in the original analysis remain so in this new version. This new version has been added to Figure 2b in the revised manuscript, replacing the original version of the analysis.

6. Only two models are applied, the saliency model than the behavioral rating model. Since the higher-level processes related to face and object recognition, are likely invariant to retinotopic location, it is not surprising that the saliency model does not explain much of the measured responses. A better prediction could be computed by first extracting the objects/faces from the stimulus images, aligning them spatially (so that eyes and mouths overlap), and then computing the model predictions. Or a Gabor filter or wavelet pyramid model could also be applied to the (spatially aligned) images. It is known that Gabor filtering model best explains the fMRI responses to faces (Carlin, Kriegeskorte, Plos Computational Biolory, 2017).

It is an interesting idea to spatially align the illusory faces in order to compute model predictions. However, it is not clear how this would be done because the variance in the location of facial features in illusory face examples is so much greater than in human faces, presenting a practical challenge to implementing this form of computational approach to our stimulus set.

For example, considering these four illusory face stimuli below, it is not clear how the illusory facial features such as “eyes” and “mouth” would be aligned to overlap across different exemplars given how different the illusory facial structure is in each case.

Note that we have substantially revised our saliency analysis in response to comments by Reviewer #2. The new analysis is shown in Figure 6 and described on pages 13-14. We also include the GIST model of visual features, which shows an extended correlation with the MEG representation over time, in comparison to the more limited correlation with the saliency model.

6

7. The main advantage of MVPA analyses is that brain responses for each individual stimulus can be compared to each other. In the decoding analyses, however, the authors collapse the data to three categories. Was it necessary to combine all samples of each category, or was the decoding also successful in individual pairs of samples or different categories?

All our analyses were based on the brain responses to individual stimuli. Although our primary focus is on the category level (i.e. what are the general effects differentiating illusory faces from non-face objects), the analyses are conducted on brain responses to individual stimuli for both fMRI and MEG:

**Decoding:** We used leave-one-exemplar-out cross decoding so that we were able to discover when (MEG) and where (fMRI) illusory faces could be discriminated from similar non-face objects. This means we did do decoding at the exemplar level (not the category level) by training the classifier on all stimulus pairs except for one held out pair on each iteration. The results were averaged to produce a category level conclusion (e.g. where/when can illusory faces be decoded from similar non face objects?). The advantage of leave-one-exemplar-out cross decoding is that although it is a much harder task for the classifier, successful decoding requires the classifier to generalize across exemplars, and thus cannot be based on particular image features of a given stimulus (Figure 2B, 5A).

**RSA:** We also show individual stimuli in the RSA analysis in Figure 2C and 5B.

We have added a new figure to the manuscript (**Supplementary Figure 3;** referenced on page 8 of main text) to show the decoding results at the exemplar level (copied below). These plots demonstrate decoding was successful at the individual stimulus level, and within all 3 categories.

|  | **New Supplementary Figure 3.** MEG decoding results at the exemplar level using multiclass classification. (a) Results for decoding all 96 stimuli using six­fold cross-validation. Chance proportion correct (1/96 = 0.01) is marked by the dashed line. Shaded area represents SEM. Multiple comparisons were controlled for using Threshold-Free Cluster Enhancement. (b) Results for decoding stimuli from the three categories (faces, illusory faces, and objects) separately using six-fold cross-validation. Chance proportion correct for each category (1/32 = 0.03) is marked by the dashed line. Shaded area represents SEM. Multiple comparisons were controlled for using Threshold-Free Cluster Enhancement. |
| --- | --- |

8. Univariate vs. multivariate responses. It would be interesting to see what the multivariate analyses add to univariate responses. Was the mean signal changes in the four ROIs similar for all three categories, or

7

did univariate results also show differences between categories. Could the authors add a notion of univariate results to the manuscript?

As suggested, we have plotted the univariate responses in the four ROIs and include it in the revised manuscript as **Supplementary Figure 2 (referenced on page 7, main text)**, along with the statistical results evaluating the differences in univariate response across the 3 stimulus categories (copied below).

**New Supplementary Figure 2.** fMRI univariate results. The mean t-value in each ROI (averaged across voxels) for each stimulus category is shown, averaged across exemplars and participants. Error bars are *SEM*. Paired sample *t*-tests (two-tailed) were conducted using the FDR correction (Benjamini and Hochberg, 1995) to control for multiple comparisons across all ROIs and category pairs. Asterisks indicate statistically significant differences, evaluated using FDR adjusted *p*-values < .05 (*), < .01 (**), and < .001 (***). Human faces elicited a stronger response than matched objects in all ROIs (FFA: *t(15) =* 7.65, *p* = 0.000009, *d* = 1.91, *95% CI*: 0.18, 0.32; OFA: *t(15) =*10.23, *p* = 0.0000004, *d* = 2.56, *95% CI*: 0.14, 0.21; PPA: *t(15) =*-4.19, *p* = 0.002, *d* = -1.05, *95% CI*: -0.20, - 0.07) except for LO (*t(15)* =-0.80, *p* = 0.43, *d* = - 0.20, *95% CI*: -0.06, 0.03). Illusory faces had a stronger response than objects in face-selective FFA (*t(15) =* 5.19, *p* = 0.0003, *d* = 1.30, *95% CI*: 0.06, 0.15) and OFA (*t(15) =* 6.91, *p* =0.00002, *d* = 1.73, *95% CI*: 0.07, 0.14), and a weaker response than objects in scene-selective PPA (*t(15) =* -2.68, *p* = 0.02, *d* = -0.67, *95% CI*: -0.06, -0.0071), with no significant difference in LO (*t(15) =* 2.03, *p* = 0.07, *d* = 0.51, *95% CI*: -0.0015, 0.06). Conversely, illusory faces produced a weaker response compared to human faces in face-selective FFA (*t(15) =* -4.75, *p* = 0.0006, *d* = -1.19, *95% CI*: -0.21, -0.08) and OFA (*t(15) =* - 3.08, *p* = 0.01, *d* = -0.7702, *95% CI*: -0.11, -0.02) and a stronger response than real faces in scene-selective PPA (*t(15) =*3.59, *p* = 0.005, *d* = 0.90, *95% CI*: 0.04, 0.16), with no significant difference in LO (*t(15) =*1.72, *p* = 0.12, *d* = 0.4303, *95% CI*: -0.01, 0.10).

9. SVM classification was used for fMRI, but LDA for MEG. What was the reason for using different method? For methodological consistency, the same method for MEG and fMRI should be preferred.

The use of different classifiers for the two neuroimaging experiments (SVM for fMRI and LDA for MEG) was planned prior to data acquisition due to their suitability for the different neuroimaging data types and their common use in the field. While both are linear classifiers, LDA is generally faster and thus suited to MEG decoding which has the added dimension of time, resulting in much longer analysis running times. Linear SVM is a standard classifier used for fMRI analysis and deals well with the issue in fMRI data of training a classifier in the case where there are many more features (voxels) compared to data points (runs).

We have added explicit justification of our classifier choice on page 24 of the Methods:

8

**“**We chose LDA for MEG decoding based on the finding that SVM and LDA produce comparable results for MEG data using the PCA preprocessing pipeline we apply here, yet LDA is generally faster and thus suited to handling MEG timeseries data (Grootswagers et al 2017).”

10. **Almost 25% percent of measured fMRI data was rejected (5/21 subjects). Four subjects were rejected due to the amount of movement. What was the criterion for rejection (how much movement), and was the criterion decided before the measurements?**

**Four subjects were rejected because of inability to define the category-selective regions (FFA, OFA, PPA, LO) from their functional localizer runs. We conducted one localizer run at the start and one at the end of each experimental session. Rejection from the final sample was based on the failure to locate the ROIs, and although this was often associated with substantial head motion producing noisy data, motion itself was not the criteria used for exclusion. We have clarified this in our statement as to why these N=4 subjects were excluded in the Methods on page 20:**

**“***N*=4 participants were removed from the fMRI analysis due to inability to define category-selective regions from their localizer data and *N*=1 was excluded due to failing to complete the experiment, leaving *N*=16 fMRI datasets for analysis.”

11. **The author emphasize the importance of yoked design, and controlling for visual features between objects and illusory faces. The real faces were not, however, yoked to the illusory faces. Is there any specific reason for this? Of course it is more difficult, but for example, the red front view of a plane could have been paired with an image of face and red clothes; green kiwi fruit with face and green clothes etc. The authors say that “Human faces were not individually matched to the illusory faces because of the ambiguity in defining parameters such as age, race and gender for illusory faces.” But colors, sizes, spatial positions, orientations can be matched.**

**Unfortunately, it is not possible to objectively yoke a particular human face to a given illusory face, thus any decision is somewhat arbitrary and there is no way to match the object content in this case. The one specific parameter we matched was the *number* of faces in the image across both illusory and human face sets (see also response to point #16 below). When considering the 'matching' of the illusory faces and human faces, we reasoned that the most important feature was to have high visual variance in both sets, since the illusory face images are highly variable -- both in the visual features that make up the illusory facial features, and in the overall color/orientation/shape etc. of each image. For this reason, we selected candid human face images “in the wild” sourced from the internet rather than from e.g. a face database, and used the same cropping procedure as we used for the illusory faces.**

**We have clarified this in the Methods on p. 21:**

**"**The illusory face images have a high degree of variance in the visual appearance of their illusory facial features, compared to human facial features. In addition, the illusory face images have greater variance in visual properties such as color, orientation, and face size, compared to the controlled face images typically used in experiments on face processing. For these reasons, the set of 32 human faces was selected to have a high degree of variance across age, gender, race, facial expression and head orientation in order to match the high variance of the illusory face image set. Human faces were not individually matched to the illusory faces because of the ambiguity in defining parameters such as age, race and gender for illusory faces. However, we did match the number of images containing more than one face across human and illusory face sets. Two of the illusory face images contained two faces, so we included two images in the human face set that also had two faces.”

9

12. While reading the manuscript, it was not always clear if statistical tests were corrected for multiple comparison and whether the same method for correction was always used. Please add, at least to figure captions, more details on statistics and corrections for multiple comparisons.

As suggested we have added additional details on statistics and controls for multiple comparisons throughout the manuscript. To summarize: we controlled for multiple comparisons in all reported statistical tests, using FDR correction for most tests (including fMRI analyses), and we used threshold-free cluster correction for the timeseries MEG analyses.

fMRI ROI decoding:

-	We have added the test statistics for the t-tests conducted on the fMRI ROI decoding analysis to page 7 in the main text including the t statistic, df, p, Cohen’s d measure of effect size, and CIs for each ROI/condition.

-	Multiple comparisons were controlled for using the FDR adjustment to the observed p-values, we have now added this detail to the text on page 7.

-	We have added the FDR correction description to the Figure 2 caption

For the new fMRI univariate analysis in Supplementary Figure 2, we used FDR (reported statistics and details are in the figure caption).

For the MEG decoding analyses and RSA model comparison, multiple comparisons were controlled using threshold-free cluster correction. We have added this detail to the caption for Figure 5 and Figure 6e and Supplementary Figure 3 .

For the new analysis testing the illusory face representations in the models of saliency/visual features in Figure 6d we used FDR correction (added to Figure 6 caption).

13. “In order to maintain attention... the image on each trial was rotated slightly to the left or right by 3°....” Does this maintain attention to stimuli, or direct attention to sides of the stimuli? Was fixation of gaze restricted center of the screen or monitored? What was the performance (mean correct) in the orientation task?

The mean correct performance on the task averaged across participants and runs was (*Mean* = 92.5%, *SD* = 8.6%) for the fMRI and (*Mean* = 93.2%, *SD* = 4.8%) for the MEG experiments respectively. We have added these results to the Figure 1 caption on page 5, and Methods page 22 (fMRI) and page 22 (MEG).

We did not intend to claim a particular type of attention was maintained by the task. The purpose of the task was to keep participants alert and looking at the centrally presented stimuli for the duration of the experiment (~1 hour MEG, ~1.5 hours fMRI). We have changed the wording from “attention” to “alertness” in the revised manuscript (page 4 (main text), Figure 1 caption, and page 21 Methods) to emphasize this.

Eye movements were not monitored. We have added this detail to the Methods on page 22 and 24. The stimuli were shown very briefly (200ms MEG, 300ms fMRI) which did not allow for substantial eye movements during stimulus presentation.

Manuscript changes:

**Results p.4**: “In order to maintain alertness, in both experiments participants judged whether the image on each trial was rotated slightly to the left or right by 3°.”

10

**Fig 1 caption:** To maintain alertness in both fMRI (*Mean* = 92.5%, *SD* = 8.6%) and MEG (*Mean* = 93.2%, *SD* = 4.8%) experiments, participants' task was to judge whether each image was tilted slightly to the left or right (3°) using a keypress.

**fMRI methods**: “A task unrelated to face detection was used in order to maintain participants’ alertness during

the fMRI experiment	Averaged across participants and runs, the mean task accuracy was 92.5% (SD = 8.6%). Eye movements were not recorded, however the brief stimulus duration prevented any substantial eye movements during stimulus presentation.”

**MEG methods:** “As for the fMRI experiment, eye movements were not recorded, however the brief stimulus duration prevented any substantial eye movements during stimulus presentation........Averaged across participants and runs, the mean task accuracy was 93.2% (*SD* = 4.8%).”

14. **The face stimuli “was selected to have a high degree of variance across age, gender, race, expression... in order to match the high variance of the illusory face..” Was this matched variance quantified somehow?**

**We did not quantify the variance in the human face stimuli, since there are not equivalent concepts such as “race” for the illusory face stimuli.**

**In the methods we state:**

“The illusory face images have a high degree of variance in the visual appearance of their illusory facial features, compared to human facial features. In addition, the illusory face images have greater variance in visual properties such as color, orientation, and face size, compared to the controlled face images typically used in experiments on face processing. For these reasons, the set of 32 human faces was selected to have a high degree of variance across age, gender, race, facial expression and head orientation in order to match the high variance of the illusory face image set. Human faces were not individually matched to the illusory faces because of the ambiguity in defining parameters such as age, race and gender for illusory faces. However, we did match the number of images containing more than one face across human and illusory face sets. Two of the illusory face images contained two faces, so we included two images in the human face set that also had two faces.” (p. 21)

**In addition, the MEG decoding results at the exemplar level (shown in the new Supp. Fig 3b; copied below) suggest that the images within each category are equivalently discriminable from the patterns of whole-brain activity. This is consistent with the images within each category being well-matched in terms of visual variance.**

15. **Behavioral rating was rating for ‘face-ness’, and ratings differ between objects and illusory faces. Did the authors consider the other way around, rating for ‘object-ness’, would illusory faces differ from objects with object-ness rating task?**

11

We selected the 32 examples of illusory faces to be nameable objects. The face ratings were collected to ensure that illusory faces were readily perceived in the examples we selected, and that faces were not perceived in the matched object images. Although “object-ness” is an interesting idea, it would be challenging to ask a comparable question since unlike faces which are a specific thing with a stereotypical structure, there is not a clear definition of what an object is, and it is a broad category that is hard to define. It is also not clear what it would tell us, as our aim was to the mechanisms underlying illusory face perception.

16. “However, we did match the number of images containing more than one face across human and illusory face sets (N = 2).” I don’t understand what the N=2 refers to, a typo? Two illusory face sets? Two images that contain more than one face? Two participants matching the stimuli?

*N*=2 referred to the number of images with more than one face in both illusory and real face sets. We have rewritten this sentence for clarity:

"However, we did match the number of images containing more than one face across human and illusory face sets. Two of the illusory face images contained two faces, so we included two images in the human face set that also had two faces.”

(p. 21, Methods)

Reviewer #2 (Remarks to the Author):

The study examines the neural profile of face detection through the joint use of fMRI and MEG as applied to illusory face perception. Specifically, it investigates the cortical locus and the temporal dynamics of pareidolia as a means to characterize the representational basis of face detection. Based on the rapid but short-lived assimilation of illusory faces to faces and to the confinement of this result to occipitotemporal face-selective regions the authors argue for a broadly-tuned template for face detection. The use of pareidolia as a window into the neural basis of face detection is certainly appealing and the results are in broad agreement with current understanding of face processing. The logic of the study is relatively straightforward and the results provide an informative contribution to the topic. My questions below mostly target aspects of the study that could benefit from further clarification.

1. The authors claim that “the representation of illusory faces was confined to occipital-temporal face-selective visual cortex” (abstract) but there is no investigation of more anterior face-selective regions (e.g., anterior temporal or inferior frontal areas). A more thorough investigation of such areas or a whole-brain analysis using searchlight could be informative here.

As suggested, we have conducted a whole-brain analysis using a decoding searchlight. We have added this analysis as a new figure in the revised manuscript (Figure 3) and describe the method on page 23 and results on page 7. The scene-face localizer we used to define FFA and OFA did not reveal an anterior face-selective region, and the searchlight decoding results suggests these ROIs captured the main regions sensitivity to illusory faces.

The results show that decoding of both human faces (a) and illusory faces (b) from matched objects is primarily in ventral temporal cortex. This analysis does not show a contribution from more anterior face-selective regions. For comparison with our planned ROI analysis, we added our functionally defined face-selective ROIs FFA and OFA on to the searchlight maps. The searchlight results indicate that the major regions of successful decoding align with FFA and OFA, with some additional area located on the ventral surface between these ROIs. This demonstrates that our planned ROIs did correspond to the main area of cortex where illusory and human faces can be distinguished from objects.

12

**New Figure 3. fMRI cross-decoding searchlight results for (a) human faces versus objects and (b) illusory faces versus objects.** For both comparisons, the location of greatest decoding accuracy is within ventral temporal cortex and overlaps with FFA particularly in the right hemisphere. The location of greatest cross-decoding for illusory faces vs matched objects (across exemplars) is a subset of the area for human faces vs. objects. For illustration, the approximate boundaries of functionally-defined FFA (purple) and OFA (green) as defined by the location of the surface nodes in individual participants (node inclusion threshold = 3/16 participants for OFA, 4/16 participants for FFA) are drawn on this example inflated surface for comparison with the searchlight results.

2. It is stated that “by 160ms, the illusory faces are more distinct from the matched objects and closer to the human faces compared to the earlier and later timepoints” (p. 9). However, an examination of Fig. 4c shows illusory faces to be less intermingled with faces at 160s (t2) compared to 130ms (t1). Please clarify.

At 160ms, the illusory faces as a whole are closer to the real faces than the matched objects are (Fig 5c) compared to the clustering at 130ms, although it is also true that they are slightly further from real faces at 160 ms compared to 130 ms.

Reviewer 1 made a similar point, and we have clarified our description of the relations between stimulus categories by revising the paragraph on page 10-11 of the Results, and adding a new paragraph to the Discussion on p. 19 which highlights how illusory faces *differ* from human faces:

p. 10-11

“The most striking feature of the representational structure is how it changes dynamically across these three

timepoints, as visualized in the dissimilarity matrices (**Fig. 5b**) and MDS plots **(Fig. 5c**). At 130 ms the human faces are already more similar to each other than to illusory faces or objects (**Fig. 5b**), as shown by the clustering of the human face exemplars in the MDS plot (**Fig. 5c**). Notably, a few illusory face exemplars are clustered with the human faces (**Fig. 5c**). By 160ms, the illusory faces have started to segregate from the matched objects (**Fig. 5c**), and have become more similar to the human faces than the matched objects are (**Fig. 5b**). There is a categorical shift in the representational organization between 160 and 260 ms: initially the illusory faces are distinct from the matched objects, but only 100 ms later, the illusory faces are grouped with the matched objects (**Fig. 5c**). As with the rapid onset of category-level cross-decoding (**Fig. 5a**), this shows that illusory faces are rapidly processed. These errors of face detection are initially treated more like real faces than matched objects are, however, the human brain rapidly resolves this detection error and they are represented more similarly to objects in less than a quarter of a second.”

p. 19

13

“Although we observe a rapid “face-like” response to illusory faces in objects, there are substantial differences in the representation of human faces versus illusory faces. The fMRI data showed that in face-selective FFA and OFA, human faces have more similar within-category activation patterns than illusory faces, which are less similar to each other. Further, in non-face areas such as LO and PPA, the illusory faces group with objects rather than human faces. This is similar in the time domain revealed by MEG, in which illusory faces have a more transient “face-like” response than human faces, and are much more similar to nonface objects within a couple hundred milliseconds. The fact that human faces and illusory faces are readily decoded from each other in both fMRI and MEG is also indicative of differences in their representation. Overall, this difference is consistent with the interpretation of illusory faces as a quickly resolved error of a broadly-tuned face detection system^2,3^. Thus although illusory faces elicit a face-like response, they are not represented in the same way as human faces within the visual system.”

3. The current use of saliency is rather problematic as a means to investigate a presumed face template. Two illusory face images could easily generate similar maps that do not represent such a template. Also, the correlation with MEG-based similarity could arise simply from low-level image similarity across illusory face images (and it would also be expected to peak early as saliency does in Fig. 5b). Perhaps a more direct test would be to correlate illusory face maps directly to face saliency maps and treat the resulting estimate(s) by analogy with face ratings in the same section/figure. This would eliminate the issue above and would also render the two analyses in Fig. 5b more immediately comparable.

We thank the reviewer for this point which made us more deeply consider what our results show with regards to saliency. With further consideration, we realize that our use of the saliency modeling to investigate the idea of a face “template” was worded more strongly in the manuscript than we intended. In response, we have substantially revised this section of the manuscript (and **Figure 6**: copied below) to clarify it conceptually. Rather than a “face template” per se, what we are interested in is how early processing of visual features may contribute to a rapid face-like response to the illusory faces.

We have replaced the phrasing “face template” with “face detection mechanism” throughout the manuscript (~7 occurrences).

We now focus on fewer models and explain them in more detail: two computational models which characterize different visual aspects of the stimuli (GBVS model of saliency, and GIST model of visual features), and the behavior model based on the face ratings.

We examine the extent to which the face-like response to illusory faces can be explained by low level visual features in two ways:

(i) Examination of how illusory faces are represented in the model RDMs for saliency and visual features. In a new analysis in Figure 6d inspired by the reviewer’s comment above, we examine the correlation between the saliency maps (for GBVS) and the feature vectors (for GIST) for all stimuli formally using a permutation test to see if the models themselves distinguish between illusory faces and matched objects. We find this is true to some extent for GIST but not for GBVS.
(ii) We compare the model RDMs for visual saliency/visual features/behavior in one figure (Fig 6e). We find that the saliency and visual feature models pick up on different aspects of the stimuli, and although both models significantly correlate with the MEG representation early on, neither computational model fully explains the behavioral or brain data.

14

|  | **New Figure 6 with revised saliency/visual feature analysis New text on p 13-15** |
| --- | --- |

4. The correlation with saliency (Supplementary Fig. 2) exhibits rather different time courses for different models: both DeepGaze2 and GIST show significance even after 400ms, unlike Itti-Koch, and GIST shows no clear peak. If the authors retain this analysis (please see point 3 above) please discuss this divergence.

As part of our extensive revision of this section (in response to the point above), we now highlight that the GBVS and GIST models show different time courses in Figure 6 (copied above) and on page 13-14. We also now include both of these models in the main text of the manuscript (Figure 6) in order to make this comparison clearer. Both the dissimilarity matrix analysis in Fig 6d and the correlation with the MEG in Fig 6e reveal that the two computational models capture different aspects of the stimuli, and while they correlate with the MEG representation to some extent, they cannot fully explain the representation revealed by the behavior.

xs

We have also addressed this point in the revised Discussion on p. 18-19:

“The early timing of the correlation between the MEG data and the saliency and visual feature models relative to that with behavior suggests that illusory face perception is at least partially driven by particular low or mid-level features. However, neither computational model fully explains the behavioral or brain data. The differences in timing reflected by the transient correlation with the GBVS saliency model and the more sustained correlation with the GIST visual feature model suggest that the two models isolate different visual characteristics that might drive the erroneous face detection response to illusory faces at different stages of processing.”

5. As the authors note, “the percept of a face persists well beyond the initial few hundred milliseconds” (p. 16). Yet, the discrimination of illusory faces from faces returns to chance before 1s, unlike the other two types of discrimination (Fig. 4a). What supports the perception of such images as ‘face-like’ at a later time?

15

**In Fig 5a, the discrimination of illusory faces from faces does not return to chance before 1s (gray line). It is the comparison between illusory face and objects that returns to chance before 1s (blue line). This is consistent with the idea that we are measuring the initial organization of these stimulus representations in visual cortex, rather than the ‘perception’ process (Discussion, page 20). We agree that it is an interesting question as to what supports the lasting perception of these stimuli as ‘face-like’, but it is outside of what we are able to interpret from the present data which instead captures the early face-like response, and reveals that it is followed by the reorganization of the stimuli as objects.**

**6. The authors use different classifiers for fMRI and MEG data, linear SVM and LDA. Presumably this is not because SVM was not successful with MEG data. Please explain.**

**The use of different classifiers (SVM for fMRI and LDA for MEG) was planned prior to data acquisition due to their suitability for the different neuroimaging data types and their common use in the field. We did not try any alternative classifiers on the fMRI or MEG datasets. See also: our response to Reviewer #1’s point #9.**

**We have added explicit justification of our classifier choice on page 24 of the Methods:**

**“We chose LDA for MEG decoding based on the finding that SVM and LDA produce comparable results for MEG data using the PCA preprocessing pipeline we apply here, yet LDA is generally faster and thus suited to handling MEG timeseries data (Grootswagers et al 2017).”**

**A few smaller issues:**

**- when displaying representational dissimilarity matrices (e.g., in Fig 4b) it could be useful to specify the**

**limits of the scales rather than just ‘min’ and ‘max’**

**As suggested we have added the limits of the colorbar scales for the RDMs for fMRI (Fig 2c) and MEG (Fig 5b) and the model-MEG figure (Fig. 6).**

**- what threshold was used for functional ROI definition and how large were the resulting ROIs (in number of voxels)?**

**We have added the size of the functional ROIs in number of voxels to the Methods section of the**

**manuscript on page 23:**

**“The mean size of each ROI averaged across participants was 271 voxels for FFA (*range: 86-577*), 190 voxels**

**for OFA (*range: 101-316*), 337 voxels for LO (*range: 70-543*), and 393 voxels for PPA *(range: 277-712*).”**

**It was not possible to use a standardized threshold across all participants for ROI definition due to differences in the range of t-values resulting from the localizer contrasts for each participant. However, we used standard procedures for defining the functional ROIs based on identifying the area of peak**

**activation for each contrast in the appropriate anatomical region of each individual participant’s structural scan as viewed on an inflated cortical surface.**

**- the current results are in relative agreement with previous studies that applied reverse correlation to derive broadly-tuned templates for face detection from MEG, EEG and fMRI signals (Smith, Fries, Gosselin et al, 2009, Cereb Cortex; Smith, Gosselin & Schyns, 2012, Curr Biol; Nestor, Vettel & Tarr, 2013, Hum Brain Mapp) and this convergence would be relevant to note**

**As suggested we have added reference to these three relevant studies in the revised manuscript, in the Discussion on pages 18-20.**

**16**

“Previous fMRI investigation of face detection in visual noise has suggested the eye and mouth regions are important features in modulating the response of face-selective visual areas, consistent with the idea of a simple template for face detection^37^”

“Our results are consistent with prior MEG results suggesting differential stages of information processing of faces that unfold over a few hundred milliseconds^39^.”

“Broadly consistent with our finding of a rapid face-like response to illusory faces, an EEG study reported an increased neuronal response across frontal and lateral occipital cortex within 500 ms to white noise patterns in which observers mistakenly thought they saw a face^42^”

**- please specify what error bars (in Fig 2b) and shaded areas (e.g., in Fig 4a) represent (standard errors, confidence intervals etc.)**

**In Fig 2b the error bars are SEM (added to Figure 2 caption)**

**In Fig 4a the shaded areas are SEM (added to caption of now Figure 5 in the revision)**

**Reviewer comments, second version:**

Reviewer #1 (Remarks to the Author):

I'm happy with the responses of the authors to all my concerns and the changes made in the manuscript. I have no further comments.


Reviewer #2 (Remarks to the Author):

The authors have addressed all my concerns.

**Author rebuttal, second version:**

**Reviewer comments, third version:**

**Author rebuttal, third version:**
